# Development of intron targeting (IT) markers specific for chromosome arm 4VS of *Haynaldia villosa* by chromosome sorting and next-generation sequencing

**DOI:** 10.1186/s12864-017-3567-z

**Published:** 2017-02-15

**Authors:** Haiyan Wang, Keli Dai, Jin Xiao, Chunxia Yuan, Renhui Zhao, Jaroslav Doležel, Yufeng Wu, Aizhong Cao, Peidu Chen, Shouzhong Zhang, Xiue Wang

**Affiliations:** 1 State Key Laboratory of Crop Genetics and Germplasm Enhancement, Cytogenetics Institute, Nanjing Agricultural University/JCIC-MCP, Nanjing, 210095 China; 2 Institute of Experimental Botany, Centre of the Haná Region for Biotechnological and Agricultural Research, Šlechtitelů 31, CZ-783671 Olomouc, Czech Republic

**Keywords:** *Triticum aestivum*, *Haynaldia villosa*, Molecular marker, Intron polymorphism

## Abstract

**Background:**

*Haynaldia villosa* (L.) Schur (syn. *Dasypyrum villosum* L. Candargy, 2n = 14, genome VV) is the tertiary gene pool of wheat, and thus a potential resource of genes for wheat improvement. Among other, wheat yellow mosaic (WYM) resistance gene *Wss1* and a take-all resistance gene were identified on the short arm of chromosome 4 V (4VS) of *H. villosa*. We had obtained introgressions on 4VS chromosome arm, with the objective of utilizing the target genes. However, monitoring these introgressions has been a daunting task because of inadequate knowledge as to *H.villosa* genome, as reflected by the lack of specific markers.

**Results:**

This study aims to develop 4VS-specific markers by combination of chromosome sorting and next-generation sequencing. The short arm of chromosome 4VS of *H.villosa* was flow-sorted using a FACSVantage SE flow cytometer and sorter, and then sequenced by Illumina sequencing. The sequence of *H. villosa* 4VS was assembled by the software Hecate, and then was compared with the sequence assemblies of wheat chromosome arms 4AL, 4BS and 4DS and *Ae. tauschii* 4DS, with the objectives of identifying exon-exon junctions and localizing introns on chromosome 4VS of *H. villosa*. The intron length polymorphisms suitable for designing *H. villosa* primers were evaluated with criteria. Consequently, we designed a total of 359 intron targeting (IT) markers, among which 232 (64.62%) markers were specific for tracing the 4VS chromatin in the wheat background.

**Conclusion:**

The combination of chromosome sorting and next-generation sequencing to develop specific IT markers for 4VS of *H. villosa* has high success rate and specificity, thus being applicable for the development of chromosome-specific markers for alien chromatin in wheat breeding.

**Electronic supplementary material:**

The online version of this article (doi:10.1186/s12864-017-3567-z) contains supplementary material, which is available to authorized users.

## Background

Wild relatives of wheat are the tertiary gene pool of bread wheat and contain many favorable genes for wheat improvement, such as disease resistance, drought tolerance, salt tolerance, winter hardiness and adaptability to poor soil [1–3]. For the utilization of these elite genes, chromosome engineering has been used to produce small alien introgressions. Irrespective of the enormous genetic variation in wild germplasm and sophisticated techniques available for alien gene transfer, it is still difficult to efficiently identify introgressed chromatin when alien introgression was used in wheat improvement.

Cytological approaches including chromosome banding, genomic in situ hybridization (GISH) and fluorescence in situ hybridization (FISH), have been extensively applied to identify and characterize introgression lines in wheat, but with limitation of low throughput [4]. Molecular markers are high efficient for identifying alien chromatin, while the numbers of molecular markers are very low in particular for the tertiary gene pool species of wheat. Hence, there is urgent need to exploit a system for high-throughput method for developing molecular markers to identify alien introgression and translocation lines.

Introns are an attractive source of polymorphism for marker development, because insertions/deletions and base substitutions are more common within intron than within exon sequences [5]. Intron length polymorphism has been considered as a convenient and reliable source of informative markers with high interspecies transferability [6]. Based on the orthologous gene conservation between rice and wheat [7], a set of PCR-based landmark unique gene (PLUG) primers were developed, and subsequently the PLUG primers were shown as being suitable to detect polymorphisms among wheat A, B and D genomes [8]. The PLUG markers not only can identify homology between wheat and alien chromosomes, thus being useful in marker assisted selection (MAS), comparative genomics, alien chromosome tracing, taxonomic studies and genotyping [9–12].

Genome mapping and sequencing in large and polyploid genomes especially wheat remain daunting tasks. However, the recent technological advances in flow cytometric sorting makes possible the dissecting of large genomes into individual chromosome, reducing sample complexity and enabling analysis at the subgenomic level [13]. Flow cytometric chromosome sorting has been successfully applied in bread and durum wheat [13]. Recently, flow-sorting of individual chromosomes has been performed in wild relatives of wheat, such as *Ae. geniculata*, *Ae. umbellulata*, *Ae. comosa*, *Ae. markgrafii*, *Ae. triuncialis*, *Ae.cylindrica*, *T. urartu*, *Ae. speltoides*, *Ae. tauschii* and *Ae. biuncialis* [14–18]. In combination with the flow sorting and the next-generation sequencing to develop DNA sequence-based markers of wild relatives of wheat have already been used to trace alien segments in wheat breeding. For example, the single-nucleotide polymorphism (SNP) markers specific for the short arm of chromosome 5M^g^S of *Aegilops geniculata* were successfully developed by chromosome sorting and next generation sequencing platform and the SNPs identified could be employed to accurately detect 5M^g^S introgressions in common wheat [14].

*Haynaldia villosa* (L.) Schur (syn. *Dasypyrum villosum* L. Candargy, 2n = 14, genome VV) is the tertiary gene pool of wheat, carrying many important genes for wheat improvement, such as resistant to several wheat diseases, including powdery mildew, eyespot, take-all, wheat spindle streak mosaic virus (WSSMV), and tolerant to drought and cold stresses, good tiller ability, and high grain protein content [19]. Previously, we identified wheat yellow mosaic resistance gene *Wss1* [20] located on the short arm of chromosome 4 V (4VS). A set of small fragment translocation lines involving 4VS were obtained using *ph1b* induction system, by which the *Wss1* was mapped to the terminal region of 4VS [21]. Here, we report an efficient method for developing chromosome-specific IT markers for the alien 4VS by chromosome sorting and next-generation sequencing, in order to utilize the beneficial genes of wild relatives for wheat breeding.

## Methods

### Plant materials

*T. durum*-*H.villosa* amphiploid (AABBVV), *T. aestivum-H. villosa* substitution line DS4V(4D), 4VS ditelosomic addition line, *T. aestivum*-*H. villosa* translocation lines T4DL · 4VS, T4VL · 4DS, NAU421, NAU428, NAU429, NAU433 and NAU435 [21]were developed at the Cytogenetics Institute, Nanjing Agricultural University (CINAU, hereafter). *H. villosa* (VV, accession No. 91C43) was obtained from Cambridge Botanical Garden, UK. Common wheat cv. Chinese Spring (AABBDD) maintained at CINAU was used as control. Chromosome composition of these materials is shown in Additional file 1: Figure S1.

### Chromosome sorting and DNA sequencing

Aqueous suspensions of chromosome 4VS of *H. villosa* were prepared from synchronized meristem root tip cells following Vrana et al. [22] and Kubaláková et al. [23, 24]. The chromosomes in suspension were stained with 2 μg/ml DAPI (4′, 6-diamidino-2-phenylindole) and the 4VS telosomes were sorted using a FACSVantage SE flow cytometer and sorter (Becton Dickinson, San Jose, USA). Purity in the sorted fractions was determined after FISH with probes for GAA microsatellite and pSc119.2 repeat on chromosomes sorted onto microscope slides. DNA of the sorted chromosome arms was purified and amplified by multiple displacement amplification (MDA) as described by Šimková et al. [25]. Three independent amplification products were combined to reduce amplification bias. The amplified DNA was purified by ethanol precipitation before sequencing.

About 10 μg of MDA-amplified DNA was used to create the two shotgun DNA-seq libraries of 500-700 bp and 700-1300 bp inserted-size. The libraries were sequenced in a single lane of Illumina HiSeq 2000 platform. The sequence read data were deposited in the (NCBI) Sequence Read Archive (SRA) and is available under accession number SRR3741672. De novo assembly of the Illumina paired-end reads was performed using the software Hecate (unpublished, http://bgi-international.com/us/) using different k-mer sizes (41, 45, 49 and 63). The result of the 45-mer run provided the assembly with the best sequence coverage and N50 size, and therefore was determined to generate 4VS scaffolds.

### Sequence resources for primer design

DNA sequences used in this work included *H. villosa* 4VS assembly, common wheat cv. Chinese Spring chromosome arms 4AL, 4BS and 4DS assemblies and annotated genes on 4DS [26] and annotated genes in the draft genome sequence assembly of *Ae. tauschii* [27].

### Homology and alignment analysis

**Fig. 1:**
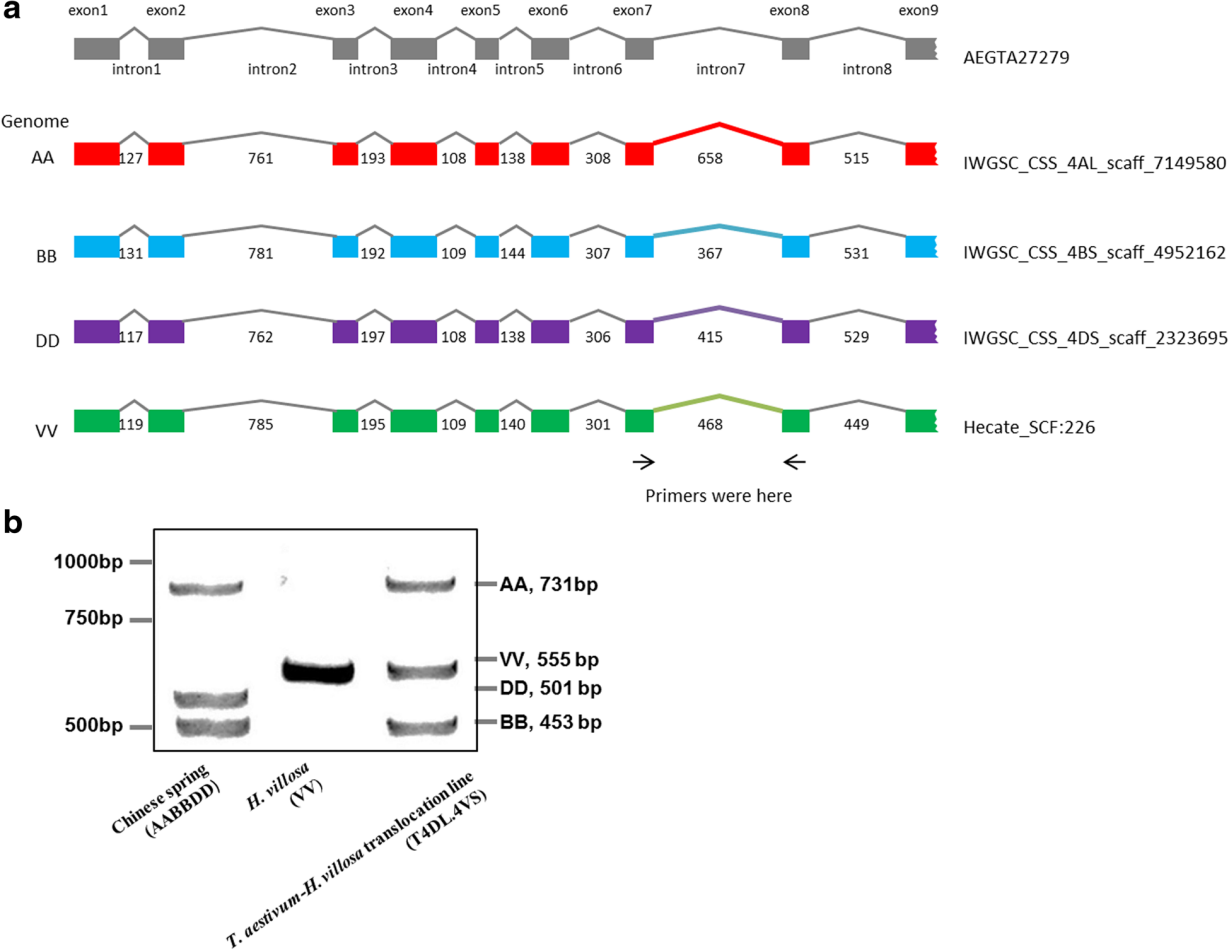
Schematic representation of the development system of PCR-based IT markers specific for the chromosome 4VS of *Haynaldia villosa* and 8% non-denaturing poly-acrylamide gels electrophoresis of PCR product of IT marker CINAU687. **a** Schematic representation of the development system of PCR-based IT markers specific for the chromosome 4VS of *Haynaldia villosa.* **b** 8% non-denaturing poly-acrylamide gels electrophoresis of PCR product of IT marker CINAU687. Grey boxes represented the exons and grey lines represented the introns. Red line, light blue line, deep blue line and light green line showed the intron 7 region of gene fragment AEGTA27279 corresponding to the intron regions of the scaffold of IWGSC_CSS_4AL_scaff_7149580, IWGSC_CSS_4BS_scaff_4952162, IWGSC_CSS_4DS_scaff_2323695 and Hecate_SCF226, respectively). The base number of intron 7 in the gene fragment of subgenome AA, BB, DD and VV was 658 bp, 367 bp, 415 bp and 468 bp, respectively

The flowchart of designing 4VS specific markers is shown in Fig. 1. In the first step, we choose a set of genes to calculate exon-exon junction sizes in genomic sequences of homoeolog arms 4AL, 4BS, 4DS and 4DS. The set contained genes from the annotated *Ae. tauschii* chromosome 4D mapped within 60 Mb of pseudomolecule and all annotated genes on Chinese Spring 4DS. We removed repeats in the two gene sets by Blastn analysis using the cut-off parameters e-value > 1e-5, coverage >80%. In the second step, all genes were compared with genomic sequences of Chinese Spring 4AL, 4BS, 4DS and *H. villosa* 4VS using a local Blastn program. All genes matching 4AL, 4BS, 4DS and 4VS assemblies and possessing at least one predicted exon-exon junction were selected. Intron sizes of corresponding genes were then calculated and compared against each other. Genes whose intron size in 4VS differed at least 10% from that of 4AL, 4BS and 4DS simultaneously in the same homologues allele were chosen for designing the markers. The primers were designed in the exons flanking the targeted introns.

### Primer design

IT primers were designed in exon regions flanking a targeted intron using the online software Primer 3 (Version 4.0, http:// frodo.wi.mit.edu/primer3/) according to the *H. villosa* sequences, assuming that exon regions and exon-intron structures of orthologous genes are highly conserved among grass species. The primers designed were expected to amplify 4AL, 4BS, 4DS and 4VS genomic DNA, simultaneously. The following criteria were used to design PCR primers for these markers: melting temperature 55–65 °C (optimum: 60 °C), primer length ranging between 18–25 bases (optimum: 20), desired size of amplified fragments estimated was approximately 50 bp more than targeted intron. All primers were synthesized by Shanghai Invitrogen Biotechnology Company Ltd. (Shanghai, China).

### DNA extraction and PCR

Genomic DNA was isolated from 2 g fresh leaves of plants at three-leaf stage with SDS-phenol-chloroform method according to Sharp et al. [28] and Devos et al. [29] and purified to eliminate RNA, amylase and other unwanted compounds. The purity and concentration of DNA was assessed with microplate reader (M200, TECan, Switzerland). The DNA was finally diluted to concentration of 50 ng/μl and stored at −20 °C until use.

PCR amplification was carried out in a 10 μl reaction volume containing 40 ng genomic DNA, 2 μM each of the primer pairs, 2.5 mM each dNTPs, 2.5 mM MgCl_2_, 1 × PCR buffer (10 mM Tris–HCl, pH 8.5, 50 mM KCl), and 0.5 U Taq DNA polymerase with a PTC-200 thermal cycler (Bio-Rad, Hercules, CA, USA). The amplification was conducted an initial denaturation at 94 °C for 3 min, followed by 35 cycles at 94 °C for 30 s, annealing of different primers at 50, 55, or 60 °C for 50 s at a ramp rate of 0.5 °C/s, 72 °C for 1 min 10 s, and a final extension at 72 °C for 10 min. PCR products were resolved in 8% non-denaturing poly-acrylamide gels (Acr : Bis = 19 : 1 or 39 : 1) and the band patterns were visualized by silver staining [30].

## Results

### Shotguns sequencing of *H. villosa* chromosome 4VS and its assembly

The analysis of DAPI-stained, chromosome suspensions prepared from a wheat-*H. villosa* 4VS addition line resulted in histograms with five peaks of fluorescence intensity (flow karyotypes). The leftmost peak represented telocentric chromosome 4VS, which was well resolved from the chromosome composite peaks I, II, III and chromosome 3B peak of the bread wheat (Additional file 2: Figure S2). The flow-sorted telocentric 4VS has more than 89% purity. DNA amplified from flow-sorted t4VS was sequenced by the Illumina technology. In total, we generated a high-quality of 33.5 Gb paired-end reads. A total length of 170.6 Mb assembled sequences was obtained, comprising 201,193 scaffolds.

### Development of PCR-based IT markers

A gene set is collected first which might have homologous alleles on 4AL, 4BS, 4DS and 4VS, simultaneously. Genes from 4VS are the first choice but poorly annotated. Genes on 4DS either from *Ae. tauschii* or common wheat, are the second choice as they are presumably most related to 4VS. *Ae. tauschii* 4DS was defined as follow. All annotated genes along the pseudomolecule of *Ae. tauschii* 4D were aligned by Blastn search against the 4VS assembly. Genes within about 60 Mb(From 0–60 Mb) had the highest frequency of Blastn hits (Data not shown). This 60-Mb interval is presumed to be *Ae. tauschii* 4DS which contained 1203 genes. A total of 1821 genes were annotated on 4DS of Chinses Spring. If one gene in Chinese Spring 4DS showed high homologous to that in the *Ae. tauschii* 4DS by Blastn analysis, they are acknowledged as the same (Additional file 3: Figure S3). All genes from *Ae. tauschii* were reserved, while redundant genes from Chinese Spring were removed, considering that the average length of genes in *Ae. tauschii* is longer than that in Chinese Spring release. Finally, we identified 1157 genes on Chinese Spring 4DS were retained. Finally, a gene set totaling 2360 genes from 4DS were established and aligned to genomic sequences of 4AL, 4BS, 4DS and 4VS using Blastn. A total of 2075 genes have blastn hits on all of four chromosome arms. Out of them, 626 genes contained at least one intron and a total of 1774 introns in the sequences of 4AL, 4BS, 4DS and 4VS with an average of 2.83 introns per gene. A total of 595 introns in 4VS differed by at least 10% as compared to those in wheat 4AL, 4BS and 4DS. They are termed “targeted intron” which meet the criteria for primer design. These 595 introns were assigned to 367 genes of which some have two or more introns. To abide “one genome marker” rule, only one such intron for each gene were selected to develop IT marker. Except for 8 genes that failed to design primers, a total of 359 primer pairs were designed which spanned the targeted intron, using the online software Primer 3 V0.4.0 (http://frodo.wi.mit.edu/primer 3/).

Take one gene for designing primers at the targeted intron for an example. The principle to develop specific IT markers of *H. villosa* 4VS was displayed in Fig. 1 *.* A gene (AEGTA27279) fragment from *Ae. tauschii* was aligned to DNA sequences of chromosome arms 4AL, 4BS, and 4DS of Chinese Spring and *H. villosa* 4VS. The gene has blastn hits on scaffolds IWGSC_CSS_4AL_scaff_7149580, IWGSC_CSS_4BS_scaff_495216, and IWGSC_CSS_4DS_scaff_2323695 on 4AL, 4BS and 4DS, and 4VS scaffold Hecate_SCF226, respectively. We compared the size of every intron of homologues alleles and found that the length of intron 7 differed among A, B, D of wheat and V genome of *H. villosa,* that they are 685 bp, 367 bp, 415 bp and 468 bp, respectively*.* IT marker was developed by designing primers on exons 7 and 8 flanking the intron 7 locus.

### Validation and the efficiency of the IT markers development by PCR

To verify the performance of the IT marker system, a total of 359 IT markers were designed using the online software Primer 3 V0.4.0 (http:// frodo.wi.mit.edu/primer3/). PCR was performed with the designed 359 primer sets using genomic DNA from the wheat cv. Chinese Spring, *T. durum*-*H. villosa* amphiploid (AABBVV), *H. villosa* and *T. aestivum*-*H. villosa* translocation line T4DL · 4VS, *T. aestivum-H. villosa* substitution line DS4V(4D), 4VS ditelosomic addition line and *T. aestivum-H. villosa* translocation line T4VL · 4DS as template. Only 14 out of the 359 primer pairs produced no amplicons, while PCR with 345 primer pairs resulted in amplicons in all four templates.

In order to assign the 345 IT markers to chromosome arm 4VS of *H. villosa*, PCR products obtained in wheat cv. Chinese Spring, *T. durum*-*H. villosa* amphiploid (AABBVV), *H. villosa* and *T. aestivum*-*H. villosa* translocation line T4DL · 4VS were separated on non-denaturing polyacrylamide gel. If the amplification of a primer pair generated a distinct PCR product shown as polymorphic band in *H. villosa*, *T. durum*-*H. villosa* amphiploid and *T. aestivum*-*H. villosa* translocation line T4DL · 4VS, but not in cv. Chinese Spring, the primer pair can be used as chromosome arm 4VS-specific marker. In total, 232 markers were found specific for 4VS (Additional file 4: Table S1). Consequently, the success rate of developing 4VS chromosome arm-specific molecular markers was as high as 64.62%.

**Fig. 2:**
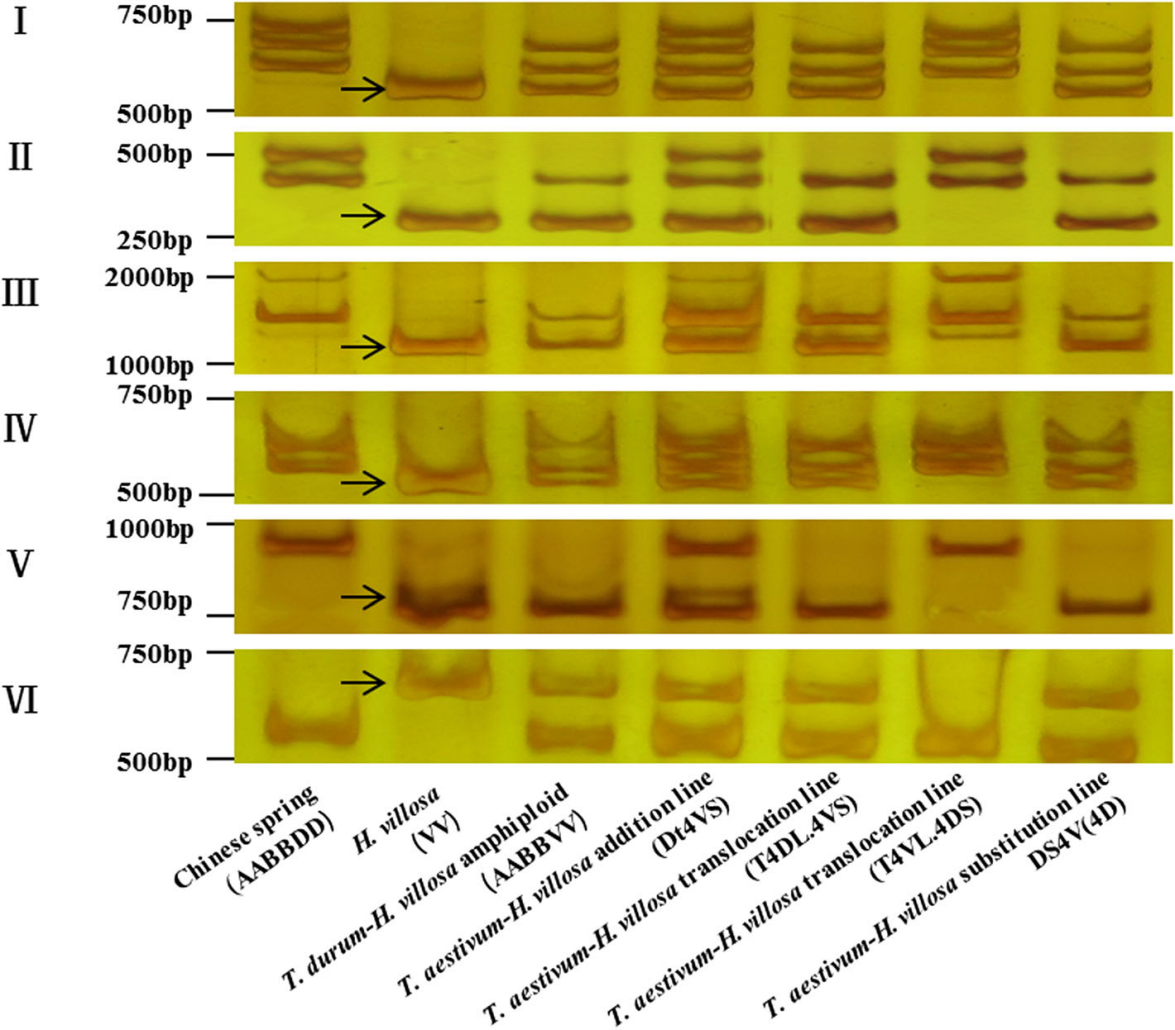
8% non-denaturing poly-acrylamide gels electrophoresis of PCR products. *Arrows* show 4VS-specific fragments

Polyacrylamide gel electrophoresis separated PCR products into two, three or four bands. Four bands were obtained with 21 primer sets (Type I), three bands with the 55 primer sets (Type II 29; Type III 5; and Type VI 21), two bands with the 156 primer sets (Type V 17 and Type VII 139) (Additional file 5: Table S2). Examples of amplification products obtained using six primer pairs are given in Fig. 2. The representative 6 types IT markers corresponding to the sequence information in subgenome AA, BB, DD and VV and the sequences of forward and reverse primers and the size of intron of the representative 6 types IT markers are given in Tables 1 and 2, respectively. The size of the products amplified by these six primer pairs ranged from 250 to 1800 bp, and each product was larger than the size predicted from the sequences of wheat and *H. villosa* intron, suggesting that all PCR products contained introns and parts of exons.

**Table 1:** The representative 6 types IT markers corresponding to the sequence information in subgenome AA, BB, DD and VV

Marker no.	Scaffold of A subgenome of Chinese Spring	Scaffold of B subgenome of Chinese Spring	Scaffold of D subgenome of Chinese Spring	Scaffold of *H.villosa*
CINAU687	IWGSC_CSS_4AL_scaff_7174192	IWGSC_CSS_4BS_scaff_4898779	IWGSC_CSS_4DS_scaff_2293539	Hecate_CTG:136974268075349775
CINAU648	IWGSC_CSS_4AL_scaff_7100654	IWGSC_CSS_4BS_scaff_4885499	IWGSC_CSS_4DS_scaff_2219420	Hecate_SCF:1351
CINAU737	IWGSC_CSS_4AL_scaff_7040796	IWGSC_CSS_4BS_scaff_4869572	IWGSC_CSS_4DS_scaff_2316979	Hecate_SCF:732
CINAU665	IWGSC_CSS_4AL_scaff_7169654	IWGSC_CSS_4BS_scaff_4884845	IWGSC_CSS_4DS_scaff_2322521	Hecate_CTG:136972502843815791
CINAU735	IWGSC_CSS_4AL_scaff_7093114	IWGSC_CSS_4BS_scaff_4873209	IWGSC_CSS_4DS_scaff_2278641	Hecate_CTG:136974259485440371
CINAU646	IWGSC_CSS_4AL_scaff_7163083	IWGSC_CSS_4BS_scaff_4906319	IWGSC_CSS_4DS_scaff_2311585	Hecate_SCF:288

**Table 2:** The sequences of forward and reverse primers and the size of intron of the representative 6 types IT markers

Marker no.	Forward primer (5’→3’)	Reverse primer (5’→3’)	Intron size of A genome	Intron size of B genome	Intron size of D genome	Intron size of V genome	Type
CINAU687	ACAGCTCATCATGCAGGACA	GTCACTGTCTTGAGCAAATGGA	632	545	687	433	TypeI
CINAU648	AGCCTCTCCTCCCTCAATCT	TGCTTCCACCTCAAATTGAACAT	269	308	281	233	TypeII
CINAU737	AAACGAGCTTTGCATGGAGG	CTTTGCATGTTGAGAAGGACAA	339	146	340	119	TypeIII
CINAU665	GCTCGGATGCAATTATTGTTGA	ATGGTCCTTCGCAGCTGTTA	639	575	576	508	TypeIV
CINAU735	TGAAGATCGTGTTCCTTCCTCT	CATGCTTTCTTCATCCCCTGG	1583	1600	939	659	TypeV
CINAU646	CATCGGTACTACGGGCGAT	TGCGGGTACTTCATCCTCAT	298	304	307	445	TypeVI

Considering the size of PCR products, the four bands amplified by the Type I markers originated from chromosomes 4A, 4B, 4D and 4VS, respectively. These 4VS-specific molecular markers developed are co-dominant and are useful to simultaneously trace the alien 4VS chromosome arm and its wheat homoeologous group.

For the Type II, Type III and Type IV markers, according to the size of introns in the subgenomes A, B, D and V, the three bands were amplified from chromosome 4D, 4A and 4 V; 4D, 4B and 4 V; 4A, 4B and 4 V, respectively. For the Type V and VII markers, the two bands detected on polyacrylamide gels consisted of the products derived from 4 VS chromosome arm, and a Chinese Spring chromosome (possibly one of 4A or 4B or 4D;or two of 4A and 4D,or 4D and 4B or 4A and 4D; or three chromosomes 4A, 4D and 4B).

Based on PCR product size polymorphisms, a total of 93 (40.09%) IT markers could be assigned to subgenomes A, B and D, 139 IT markers could not be assigned to subgenomes A, B and D. We compared the intron sizes of subgenomes A, B and D and found that the difference in the intron size between homoelogs was small.

## Discussion

Wild relatives of wheat contain a large number of favorable genes for resistance to biotic and abiotic stresses and can be used for wheat improvement. But now, monitoring alien introgressions in wheat background is difficult because of the shortage of genetic and molecular mapping information on the wild relatives. DNA markers can detect small amounts of alien chromatin that cannot be recognized cytogenetically, thus having high efficiency in the identification of the introgressed fragments. Simple sequence repeat (SSR) was used as the molecular markers to screen the individual chromosome of wild relatives of wheat. However, they were low in transferability to wild relatives and were lack in locus specificity, and therefore had limitation in application to wheat breeding [31]. Taken the *H. villosa* as an example, only 9 of the 276 wheat microsatellites had high polymorphisms suitable for molecular markers [32]. Random amplified polymorphic DNAs (RAPD), although easy to develop, still suffer from poor reproducibility [33]. Bin-mapped expressed sequence tags (ESTs) were also explored as a source of markers, but only a few of them were polymorphic [34]. Zhao et al. [35] designed 607 primer pairs from wheat EST sequences and found that only 58 (9.23%) of the primers amplified specific bands from chromosome 4 V. Up to now, there is still fewer molecular markers specific for individual chromosome of wild relatives that can be used, this is partially due to the lack of genome information of the relatives, as reflected by the fact that most of the markers were designed on the basis of genome of wheat, rice, or other species.

Flow cytometric chromosome sorting is an efficient method for dramatically simplifying genome analysis by reducing DNA sequence complexity [36]. And it combination with next-generation sequencing technology could obtain the sequence composition of single chromosome of wild relatives, thus being highly productive in development and validation of molecular markers [36]. As next-generation sequencing technologies become more economical, making possible the high throughput mining of molecular markers specific for chromosome. Moreover, all wheat chromosomes have been sequenced using flow sorting and next-generation sequencing, which should be useful for the developing molecular markers specific for alien chromosome [26]. Tiwari et al. [14] developed a total of 2178 unique, 5M^g^S-specific SNPs of the *Ae. geniculata* by the combination of chromosome sorting and the next generation sequencing platform, and showed that this approach has high-throughput for the discovery of markers specific for wild relative. In the present study, we first flow-sorted the chromosome arm 4VS of *H. villosa*, thereby drastically reducing the amount of work on DNA sequencing of this wild relatives. Based on the genome sequence of 4VS, we designed IT markers, which are based on the sequence conservation of orthologous genes and therefore have higher transferability between Triticeae species. A total of 359 IT markers were designed and 232 (64.62%) markers proved to be specific for *H. villosa* 4VS. Compared to RAPD, SSR, and EST-PCR markers developed by conventional methods [32, 33, 35], the IT markers designing by chromosome sorting and next-generation sequencing is more efficient with high success rate and specificity.

In our lab, Zhao et al. used wheat *ph1b* mutant to induce translocations of 4VS chromosome fragments to further physically map the *Wss1* to specific chromosome region [21]. However, due to the lower density of markers used in determination of the translocated fragments, a limited resolution of physical maps consisting of 13 bins were obtained. The IT markers developed in this study will dramatically increase the density of 4VS physical map or cytological map using these structural aberrants involving 4VS chromosome. The development of a large number of IT markers will be invaluable to trace alien chromatin in a wheat background, for comparative genome mapping, chromosomal evolutionary analysis, and gene introgression during wheat improvement using *H. villosa* as gene donor. Twenty-one 4VS-specific molecular markers developed in this work are co-dominant and are useful to simultaneously trace the alien 4VS chromosome arm and its wheat homoeologues.

In the present research, we randomly used 100 specific IT markers for the physical mapping analysis and tracing alien chromatin in wheat background. Using template DNA from five wheat-*H. villosa* 4VS translocation lines (NAU421, NAU428, NAU429, NAU433 and NAU435) and wheat-*H. villosa* T4DL · 4VS translocation line, we examined the presence or absence of the 100 specific IT markers. Thus, the 18, 55, 69, 24 and 44 IT markers were assigned to the region of wheat-*H. villosa* 4VS translocation lines (NAU421, NAU428, NAU429, NAU433 and NAU435), respectively(Additional file 6: Table S3).

Wheat spindle streak mosaic virus (WSSMV) and wheat yellow mosaic virus (WYMV) resistance gene *Wss1* were previously mapped to the 4VS arm of *H. villosa*, using 4 V (4D) substitution and T4DL · 4VS translocation lines [20]. For a more accurate mapping of *Wss1* gene, a *ph1b* mutant of cv. Chinese Spring was used to induce new translocations with smaller 4VS chromosome fragments. Based on the resistance evaluation, GISH and molecular marker analysis of the available translocations, the gene(s) conferring the WYMV resistance on 4VS were mapped to the distal region of 4VS in the bin of FL 0.78–1.00 [21]. If the resistance genes are fully explored and used, they would greatly enrich the available resistance germplasm resources for wheat. Wheat-*H. villosa* 4VS translocation lines (NAU421, NAU428, NAU429) contained wheat yellow mosaic virus (WYMV) resistance gene, *Wss1*. So these IT markers located in the terminal region will be helpful for marker-assisted introgression of the genes of interest, such as *Wss1* gene, into elite cultivars of the common wheat.

Miftahudin et al. [37] demonstrated that chromosome 4A have undergone two reciprocal translocations and two inversions events that placed most of the ancestral short arm on the modern long arm (4AL). In our lab, we also used ChromoWiz to define the 4VS syntenic regions in wheat chromosomes and found that they were enriched on wheat group 4 chromosomes 4AL. To develop 4VS-specific IT marker, we used DNA sequences obtained from flow-sorted chromosome arm 4VS of *H. villosa* was compared with the sequence assemblies of wheat homoeologous group 4 chromosomes (4AL, 4BS and 4DS) and *Ae. tauschii* 4DS to identify exon-exon junctions and localize introns on chromosome 4VS of *H. villosa*.

## Conclusions

In this study, we flow-sorted a ditelosomic addition wheat-*H. villosa* line to isolate the short arm of the *H.villosa* 4VS chromosome with ~89% purity identified by FISH using a FACSVantage SE flow cytometer and sorter. This approach reduced DNA sample complexity and permitted the development of markers specific for the short arm of 4VS. The sequence of *H. villosa* 4VS was assembled by the software Hecate, and then was compared with the sequence assemblies of wheat homoeologous group 4 chromosomes and *Ae. tauschii* 4DS to identify exon-exon junctions and localize introns on chromosome 4VS of *H. villosa*. The intron length polymorphisms suitable for designing *H. villosa* primers were evaluated with criteria, whose intron size of genes in 4VS differed at least 10% from that of 4AL, 4BS and 4DS simultaneously in the same homologues allele were chosen for designing the markers. Lastly, we designed a total of 359 IT markers, among which 232 (64.62%) markers were found specific for 4VS, with the success rate being as high as 64.62%. Collectively, this approach of combination of chromosome sorting and genome sequencing can be applicable for development of species/genome-specific markers to trace alien chromatin in wheat background, or for comparative genome mapping, chromosomal evolutionary analysis.

## Additional files

Additional file 1: Figure S1. The chromosome composition of these materials used in the experiment. (PNG 138 kb)
Additional file 2: Figure S2. Histogram of relative DAPI fluorescence (flow karyotype). Histogram obtained after flow cytometric analysis of mitotic metaphase chromosomes of T. aestivum-H. villosa ditelosomic additional line Dt4VS. Peak corresponding to telosomes 4VS (red arrow pointed) is well discriminated, which facilitated their flow sorting. Sorted chromosome arms were identified after FISH with probes for GAA (green) and pSc119.2 (red) repeat, which results in characteristic banding pattern (inset). X-axis: relative DAPI fluorescence intensity; Y-axis: number of particles. (PNG 232 kb)
Additional file 3: Figure S3. The numbers of genes annotated in Chinese Spring 4DS chromosome and 60-Mb of *Ae. tauschii* 4D. The Venn diagrams displayed the numbers of genes differently annotated in Chinese Spring 4DS chromosome and 60-Mb of *Ae. tauschii* 4D (outer cycle), and the number of shared conserved genes among the two chromosomes (inner cycle). (PNG 133 kb)
Additional file 4: Table S1. The IT markers corresponding to the sequence information in subgenome AA, BB, DD and VV (DOCX 38 kb)
Additional file 5: Table S2. The sequences of forward and reverse primers and the size of intron of the IT markers (DOCX 48 kb)
Additional file 6: Table S3. PCR products of 100 IT markers. (DOCX 101 kb)

## Abbreviations

ESTs: Expressed sequence tags
FISH: Fluorescence *in situ* hybridization
GISH: Genomic *in situ* hybridization
IT: Intron targeting
MAS: Marker assisted selection
PLUG: PCR-based landmark unique gene
RAPD: Random amplified polymorphic DNAs
SNP: Single-nucleotide polymorphism
SSR: Simple sequence repeat
WSSMV: Wheat spindle streak mosaic virus
WYMV: Wheat yellow mosaic virus.
