# Supplementary figures and images for: Development of intron targeting (IT) markers specific for chromosome arm 4VS of Haynaldia villosa by chromosome sorting and next-generation sequencing
Source: BMC Genomics. 2017 Feb 15;18:167. doi: 10.1186/s12864-017-3567-z (PMC5310052; doi:10.1186/s12864-017-3567-z)

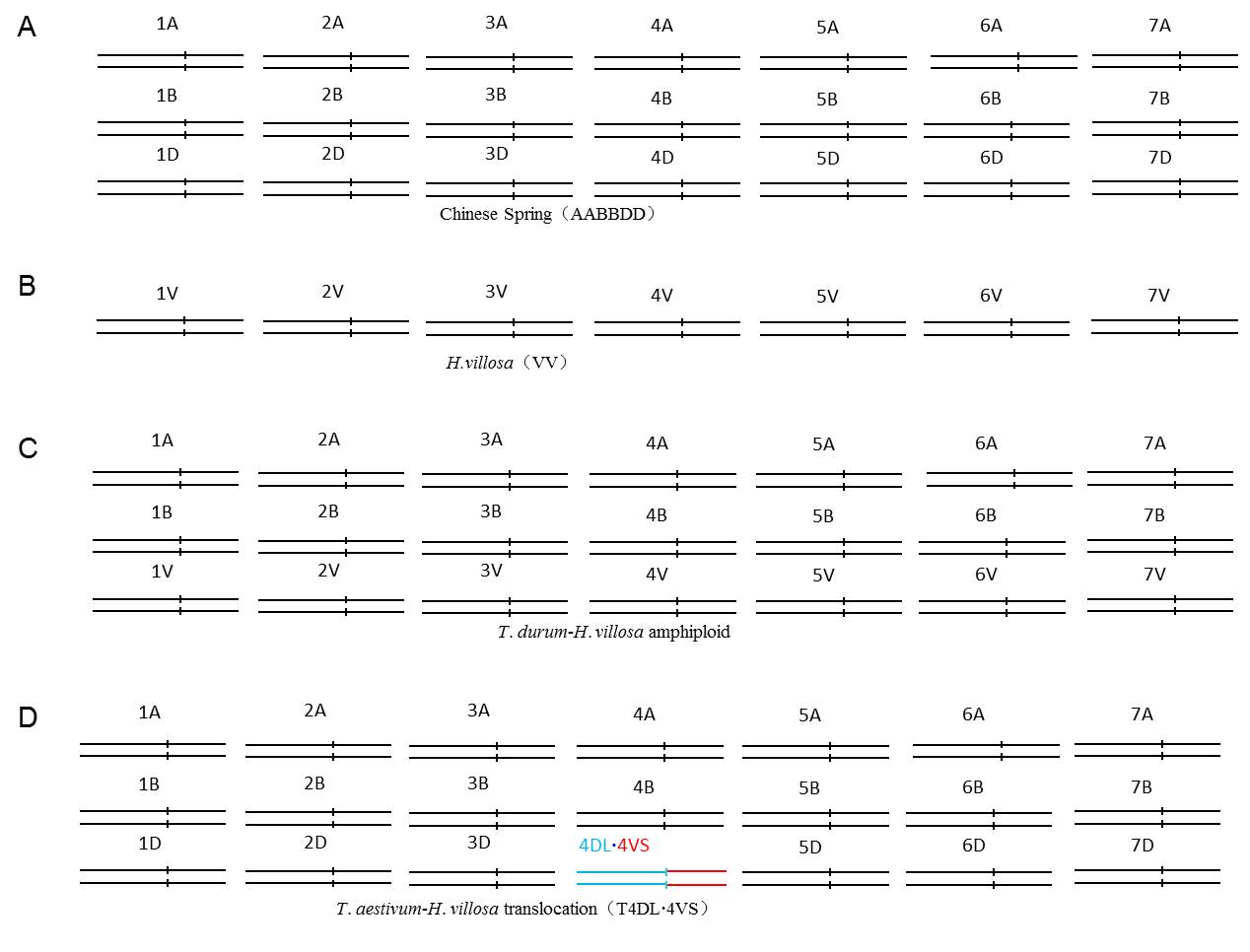

Supplement: Additional file 1: Figure S1. — The chromosome composition of these materials used in the experiment. (PNG 138 kb) [file 12864_2017_3567_MOESM1_ESM.png]

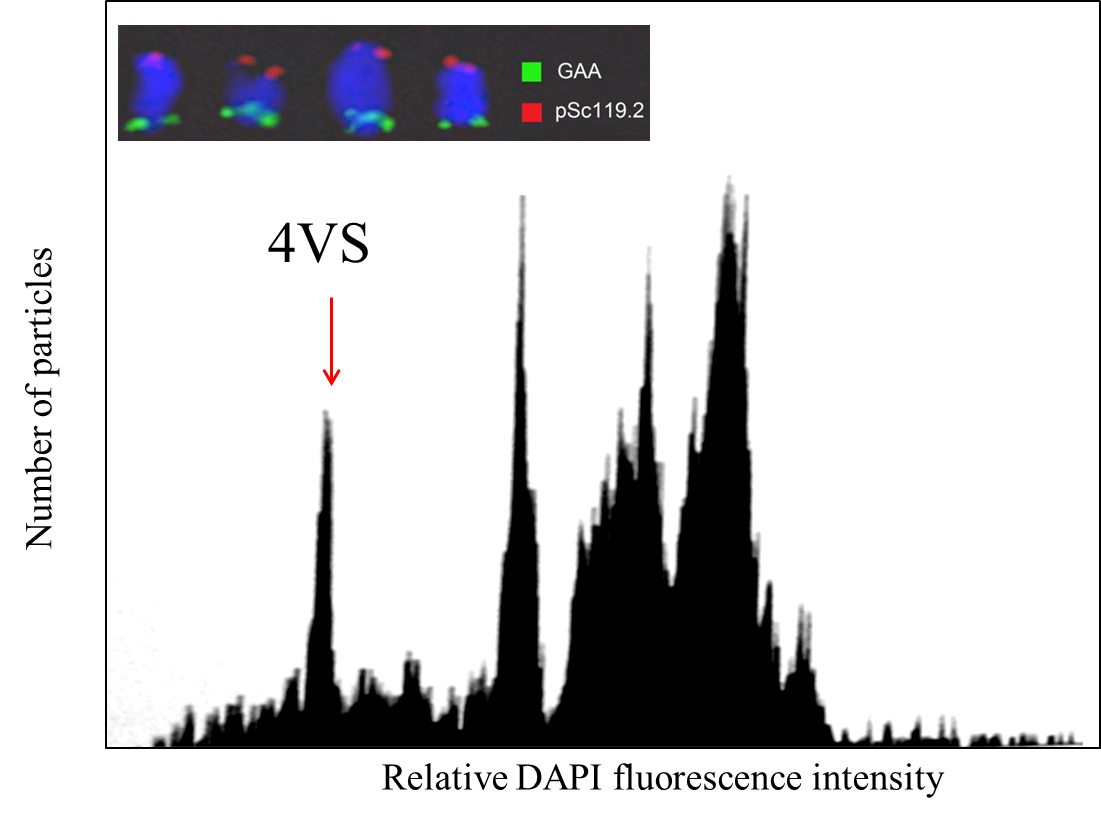

Supplement: Additional file 2: Figure S2. — Histogram of relative DAPI fluorescence (flow karyotype). Histogram obtained after flow cytometric analysis of mitotic metaphase chromosomes of T. aestivum-H. villosa ditelosomic additional line Dt4VS. Peak corresponding to telosomes 4VS (red arrow pointed) is well discriminated, which facilitated their flow sorting. Sorted chromosome arms were identified after FISH with probes for GAA (green) and pSc119.2 (red) repeat, which results in characteristic banding pattern (inset). X-axis: relative DAPI fluorescence intensity; Y-axis: number of particles. (PNG 232 kb) [file 12864_2017_3567_MOESM2_ESM.png]

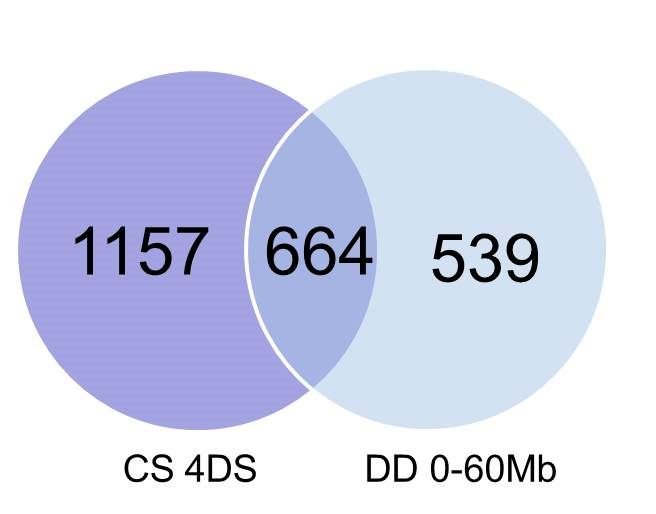

Supplement: Additional file 3: Figure S3. — The numbers of genes annotated in Chinese Spring 4DS chromosome and 60-Mb of Ae. tauschii 4D. The Venn diagrams displayed the numbers of genes differently annotated in Chinese Spring 4DS chromosome and 60-Mb of Ae. tauschii 4D (outer cycle), and the number of shared conserved genes among the two chromosomes (inner cycle). (PNG 133 kb) [file 12864_2017_3567_MOESM3_ESM.png]
