# Supplementary material for: Development of intron targeting (IT) markers specific for chromosome arm 4VS of Haynaldia villosa by chromosome sorting and next-generation sequencing
Source: BMC Genomics. 2017 Feb 15;18:167. doi: 10.1186/s12864-017-3567-z (PMC5310052; doi:10.1186/s12864-017-3567-z)
Supplement: Additional file 4: Table S1. — The IT markers corresponding to the sequence information in subgenome AA, BB, DD and VV (DOCX 38 kb) [file 12864_2017_3567_MOESM4_ESM.docx]

| Marker no.  Table S1 The IT markers corresponding to the sequence information in subgenome AA, BB, DD and VV | Scaffold of A subgenome of Chinese Spring | Scaffold of B subgenome of Chinese Spring | Scaffold of D subgenome of Chinese Spring | Scaffold of *H.villosa* |
| --- | --- | --- | --- | --- |
| CINAU604 | IWGSC_CSS_4AL_scaff_7114387 | IWGSC_CSS_4BS_scaff_1280055 | IWGSC_CSS_4DS_scaff_2308408 | Hecate_CTG:136973864348473775 |
| CINAU605 | IWGSC_CSS_4AL_scaff_7119138 | IWGSC_CSS_4BS_scaff_4959033 | IWGSC_CSS_4DS_scaff_2318442 | Hecate_SCF:240 |
| CINAU606 | IWGSC_CSS_4AS_scaff_5189300 | IWGSC_CSS_4BS_scaff_4945549 | IWGSC_CSS_4DL_scaff_14427279 | Hecate_CTG:136974620262712103 |
| CINAU607 | IWGSC_CSS_4AL_scaff_7108630 | IWGSC_CSS_4BS_scaff_4959522 | IWGSC_CSS_4DS_scaff_2326444 | Hecate_SCF:586 |
| CINAU608 | IWGSC_CSS_4AL_scaff_7078584 | IWGSC_CSS_4BS_scaff_4899184 | IWGSC_CSS_4DS_scaff_1907444 | Hecate_SCF:603 |
| CINAU609 | IWGSC_CSS_4AL_scaff_3181030 | IWGSC_CSS_4BS_scaff_4870233 | IWGSC_CSS_4DS_scaff_2320281 | Hecate_CTG:136974160701220671 |
| CINAU610 | IWGSC_CSS_4AL_scaff_7094586 | IWGSC_CSS_4BS_scaff_4886078 | IWGSC_CSS_4DS_scaff_2277109 | Hecate_SCF:1351 |
| CINAU611 | IWGSC_CSS_4AL_scaff_7170952 | IWGSC_CSS_4BS_scaff_4960341 | IWGSC_CSS_4DS_scaff_2300940 | Hecate_CTG:136973108434180519 |
| CINAU612 | IWGSC_CSS_4AL_scaff_3389281 | IWGSC_CSS_4BS_scaff_4930473 | IWGSC_CSS_4DS_scaff_2305402 | Hecate_CTG:136973263053006727 |
| CINAU613 | IWGSC_CSS_4AL_scaff_7069482 | IWGSC_CSS_4BS_scaff_4870233 | IWGSC_CSS_4DS_scaff_2316712 | Hecate_CTG:136974418399258325 |
| CINAU614 | IWGSC_CSS_4AL_scaff_7095962 | IWGSC_CSS_4BS_scaff_4890614 | IWGSC_CSS_4DS_scaff_1474230 | Hecate_SCF:1072 |
| CINAU615 | IWGSC_CSS_4AL_scaff_7095962 | IWGSC_CSS_4BS_scaff_4890614 | IWGSC_CSS_4DS_scaff_1474230 | Hecate_SCF:1072 |
| CINAU616 | IWGSC_CSS_4AL_scaff_7130147 | IWGSC_CSS_4BS_scaff_4959618 | IWGSC_CSS_4DS_scaff_2279114 | Hecate_SCF:5531 |
| CINAU617 | IWGSC_CSS_4AL_scaff_7079971 | IWGSC_CSS_4BS_scaff_4952162 | IWGSC_CSS_4DS_scaff_2323695 | Hecate_SCF:274 |
| CINAU618 | IWGSC_CSS_4AL_scaff_7079971 | IWGSC_CSS_4BS_scaff_4952162 | IWGSC_CSS_4DS_scaff_2323695 | Hecate_SCF:226 |
| CINAU619 | IWGSC_CSS_4AL_scaff_7175574 | IWGSC_CSS_4BS_scaff_4953173 | IWGSC_CSS_4DS_scaff_2277470 | Hecate_SCF:2664 |
| CINAU620 | IWGSC_CSS_4AL_scaff_7085956 | IWGSC_CSS_4BS_scaff_4961701 | IWGSC_CSS_4DS_scaff_1947998 | Hecate_SCF:1585 |
| CINAU621 | IWGSC_CSS_4AS_scaff_5985544 | IWGSC_CSS_4BL_scaff_7002103 | IWGSC_CSS_4DL_scaff_14407694 | Hecate_CTG:136973911593073401 |
| CINAU622 | IWGSC_CSS_4AL_scaff_7102797 | IWGSC_CSS_4BS_scaff_4921961 | IWGSC_CSS_4DS_scaff_2304139 | Hecate_SCF:1753 |
| CINAU623 | IWGSC_CSS_4AS_scaff_5959988 | IWGSC_CSS_4BS_scaff_4923312 | IWGSC_CSS_4DS_scaff_2299550 | Hecate_SCF:217 |
| CINAU624 | IWGSC_CSS_4AL_scaff_7063259 | IWGSC_CSS_4BS_scaff_4861837 | IWGSC_CSS_4DS_scaff_2303712 | Hecate_SCF:3746 |
| CINAU625 | IWGSC_CSS_4AL_scaff_7117336 | IWGSC_CSS_4BS_scaff_4898691 | IWGSC_CSS_4DS_scaff_2323195 | Hecate_SCF:2065 |
| CINAU626 | IWGSC_CSS_4AL_scaff_7157602 | IWGSC_CSS_4BS_scaff_4910453 | IWGSC_CSS_4DS_scaff_2326263 | Hecate_SCF:346 |
| CINAU627 | IWGSC_CSS_4AL_scaff_7066394 | IWGSC_CSS_4BS_scaff_4873206 | IWGSC_CSS_4DS_scaff_2278504 | Hecate_CTG:136974444169044739 |
| CINAU628 | IWGSC_CSS_4AL_scaff_7092406 | IWGSC_CSS_4BS_scaff_4869752 | IWGSC_CSS_4DS_scaff_2279815 | Hecate_CTG:136973950247768033 |
| CINAU629 | IWGSC_CSS_4AL_scaff_7149418 | IWGSC_CSS_4BS_scaff_4927328 | IWGSC_CSS_4DS_scaff_2282005 | Hecate_SCF:1000 |
| CINAU630 | IWGSC_CSS_4AL_scaff_7173434 | IWGSC_CSS_4BS_scaff_4923393 | IWGSC_CSS_4DS_scaff_2316777 | Hecate_SCF:949 |
| CINAU631 | IWGSC_CSS_4AL_scaff_7080509 | IWGSC_CSS_4BS_scaff_4901537 | IWGSC_CSS_4DS_scaff_1231785 | Hecate_SCF:838 |
| CINAU632 | IWGSC_CSS_4AL_scaff_7162344 | IWGSC_CSS_4BS_scaff_4874366 | IWGSC_CSS_4DS_scaff_2279841 | Hecate_CTG:136973275937953547 |
| CINAU633 | IWGSC_CSS_4AS_scaff_5938792 | IWGSC_CSS_4BS_scaff_4955888 | IWGSC_CSS_4DS_scaff_2301348 | Hecate_CTG:136973104139252265 |
| CINAU634 | IWGSC_CSS_4AL_scaff_2555292 | IWGSC_CSS_4BS_scaff_4883884 | IWGSC_CSS_4DS_scaff_2296575 | Hecate_SCF:644 |
| CINAU635 | IWGSC_CSS_4AL_scaff_7158457 | IWGSC_CSS_4BS_scaff_4946439 | IWGSC_CSS_4DS_scaff_2295679 | Hecate_SCF:2444 |
| CINAU636 | IWGSC_CSS_4AL_scaff_7120991 | IWGSC_CSS_4BS_scaff_4920865 | IWGSC_CSS_4DS_scaff_2295679 | Hecate_SCF:443 |
| CINAU637 | IWGSC_CSS_4AL_scaff_4064682 | IWGSC_CSS_4BS_scaff_4912297 | IWGSC_CSS_4DS_scaff_2275675 | Hecate_SCF:640 |
| CINAU638 | IWGSC_CSS_4AL_scaff_7080772 | IWGSC_CSS_4BS_scaff_4914659 | IWGSC_CSS_4DS_scaff_2276823 | Hecate_SCF:382 |
| CINAU639 | IWGSC_CSS_4AL_scaff_7125200 | IWGSC_CSS_4BS_scaff_4933728 | IWGSC_CSS_4DS_scaff_2277956 | Hecate_SCF:1496 |
| CINAU640 | IWGSC_CSS_4AL_scaff_7154345 | IWGSC_CSS_4BS_scaff_4883119 | IWGSC_CSS_4DS_scaff_2289475 | Hecate_CTG:136974731931847811 |
| CINAU641 | IWGSC_CSS_4AL_scaff_7154345 | IWGSC_CSS_4BS_scaff_4883119 | IWGSC_CSS_4DS_scaff_2289475 | Hecate_CTG:136974731931847811 |
| CINAU642 | IWGSC_CSS_4AL_scaff_7073251 | IWGSC_CSS_4BS_scaff_4961723 | IWGSC_CSS_4DS_scaff_2300477 | Hecate_CTG:136973194333571635 |
| CINAU643 | IWGSC_CSS_4AL_scaff_7135849 | IWGSC_CSS_4BS_scaff_4923101 | IWGSC_CSS_4DS_scaff_2306138 | Hecate_SCF:698 |
| CINAU644 | IWGSC_CSS_4AL_scaff_7089608 | IWGSC_CSS_4BS_scaff_4883210 | IWGSC_CSS_4DS_scaff_2302593 | Hecate_SCF:2682 |
| CINAU645 | IWGSC_CSS_4AL_scaff_7103176 | IWGSC_CSS_4BS_scaff_4933821 | IWGSC_CSS_4DS_scaff_2325863 | Hecate_SCF:1828 |
| CINAU646 | IWGSC_CSS_4AL_scaff_7163083 | IWGSC_CSS_4BS_scaff_4906319 | IWGSC_CSS_4DS_scaff_2311585 | Hecate_SCF:288 |
| CINAU647 | IWGSC_CSS_4AL_scaff_7104354 | IWGSC_CSS_4BS_scaff_4875860 | IWGSC_CSS_4DS_scaff_2286364 | Hecate_CTG:136972253735722503 |
| CINAU648 | IWGSC_CSS_4AL_scaff_7100654 | IWGSC_CSS_4BS_scaff_4885499 | IWGSC_CSS_4DS_scaff_2219420 | Hecate_SCF:1351 |
| CINAU649 | IWGSC_CSS_4AL_scaff_7067843 | IWGSC_CSS_4BS_scaff_4939708 | IWGSC_CSS_4DS_scaff_2286750 | Hecate_SCF:1817 |
| CINAU650 | IWGSC_CSS_4AL_scaff_7067843 | IWGSC_CSS_4BS_scaff_4939708 | IWGSC_CSS_4DS_scaff_2286750 | Hecate_SCF:1817 |
| CINAU651 | IWGSC_CSS_4AL_scaff_7139664 | IWGSC_CSS_4BS_scaff_4871016 | IWGSC_CSS_4DS_scaff_2317970 | Hecate_SCF:1576 |
| CINAU652 | IWGSC_CSS_4AL_scaff_7075566 | IWGSC_CSS_4BS_scaff_4161517 | IWGSC_CSS_4DS_scaff_2294992 | Hecate_CTG:136972253735736115 |
| CINAU653 | IWGSC_CSS_4AL_scaff_7137551 | IWGSC_CSS_4BS_scaff_4895885 | IWGSC_CSS_4DS_scaff_2311943 | Hecate_CTG:136972730477106347 |
| CINAU654 | IWGSC_CSS_4AL_scaff_7137551 | IWGSC_CSS_4BS_scaff_4895885 | IWGSC_CSS_4DS_scaff_2311943 | Hecate_SCF:1809 |
| CINAU655 | IWGSC_CSS_4AL_scaff_7120959 | IWGSC_CSS_4BS_scaff_755812 | IWGSC_CSS_4DS_scaff_2320707 | Hecate_CTG:136974607377793055 |
| CINAU656 | IWGSC_CSS_4AL_scaff_7067278 | IWGSC_CSS_4BS_scaff_4962839 | IWGSC_CSS_4DS_scaff_2313916 | Hecate_SCF:2566 |
| CINAU657 | IWGSC_CSS_4AL_scaff_7073758 | IWGSC_CSS_4BS_scaff_4892270 | IWGSC_CSS_4DS_scaff_2322828 | Hecate_SCF:705 |
| CINAU658 | IWGSC_CSS_4AL_scaff_7064781 | IWGSC_CSS_4BS_scaff_4884666 | IWGSC_CSS_4DS_scaff_244205 | Hecate_SCF:1753 |
| CINAU659 | IWGSC_CSS_4AL_scaff_7111025 | IWGSC_CSS_4BS_scaff_4909671 | IWGSC_CSS_4DS_scaff_2324459 | Hecate_SCF:288 |
| CINAU660 | IWGSC_CSS_4AL_scaff_7126077 | IWGSC_CSS_4BS_scaff_4874440 | IWGSC_CSS_4DS_scaff_2326610 | Hecate_SCF:632 |
| CINAU661 | IWGSC_CSS_4AL_scaff_7033571 | IWGSC_CSS_4BL_scaff_7037291 | IWGSC_CSS_4DS_scaff_2289149 | Hecate_CTG:136975178608425835 |
| CINAU662 | IWGSC_CSS_4AL_scaff_7174213 | IWGSC_CSS_4BS_scaff_4901448 | IWGSC_CSS_4DS_scaff_2316344 | Hecate_SCF:465 |
| CINAU663 | IWGSC_CSS_4AL_scaff_7139376 | IWGSC_CSS_4BS_scaff_3154277 | IWGSC_CSS_4DS_scaff_2312826 | Hecate_CTG:136973297412779727 |
| CINAU664 | IWGSC_CSS_4AL_scaff_7145348 | IWGSC_CSS_4BS_scaff_4944346 | IWGSC_CSS_4DS_scaff_2316170 | Hecate_SCF:3624 |
| CINAU665 | IWGSC_CSS_4AL_scaff_7169654 | IWGSC_CSS_4BS_scaff_4884845 | IWGSC_CSS_4DS_scaff_2322521 | Hecate_CTG:136972502843815791 |
| CINAU666 | IWGSC_CSS_4AL_scaff_7063814 | IWGSC_CSS_4BS_scaff_4879245 | IWGSC_CSS_4DS_scaff_2310542 | Hecate_SCF:2312 |
| CINAU667 | IWGSC_CSS_4AL_scaff_7063814 | IWGSC_CSS_4BS_scaff_4879245 | IWGSC_CSS_4DS_scaff_2310542 | Hecate_SCF:2312 |
| CINAU668 | IWGSC_CSS_4AL_scaff_7169311 | IWGSC_CSS_4BS_scaff_4949658 | IWGSC_CSS_4DS_scaff_2295334 | Hecate_SCF:3427 |
| CINAU669 | IWGSC_CSS_4AL_scaff_7169311 | IWGSC_CSS_4BS_scaff_4949658 | IWGSC_CSS_4DS_scaff_2295334 | Hecate_SCF:3427 |
| CINAU670 | IWGSC_CSS_4AL_scaff_7118584 | IWGSC_CSS_4BS_scaff_4925058 | IWGSC_CSS_4DS_scaff_2309698 | Hecate_CTG:136972855031125063 |
| CINAU671 | IWGSC_CSS_4AL_scaff_7118584 | IWGSC_CSS_4BS_scaff_4925666 | IWGSC_CSS_4DS_scaff_2309698 | Hecate_SCF:1187 |
| CINAU672 | IWGSC_CSS_4AL_scaff_7136329 | IWGSC_CSS_4BS_scaff_4917938 | IWGSC_CSS_4DS_scaff_2306417 | Hecate_SCF:5425 |
| CINAU673 | IWGSC_CSS_4AL_scaff_7171255 | IWGSC_CSS_4BS_scaff_4952268 | IWGSC_CSS_4DS_scaff_2324686 | Hecate_SCF:1351 |
| CINAU674 | IWGSC_CSS_4AL_scaff_7176580 | IWGSC_CSS_4BS_scaff_4959688 | IWGSC_CSS_4DS_scaff_2322509 | Hecate_SCF:497 |
| CINAU675 | IWGSC_CSS_4AL_scaff_7174597 | IWGSC_CSS_4BS_scaff_4907847 | IWGSC_CSS_4DS_scaff_2306944 | Hecate_SCF:619 |
| CINAU676 | IWGSC_CSS_4AL_scaff_7133091 | IWGSC_CSS_4BS_scaff_4959110 | IWGSC_CSS_4DS_scaff_2324926 | Hecate_SCF:2330 |
| CINAU677 | IWGSC_CSS_4AL_scaff_7080866 | IWGSC_CSS_4BS_scaff_4917015 | IWGSC_CSS_4DS_scaff_2283386 | Hecate_SCF:140 |
| CINAU678 | IWGSC_CSS_4AL_scaff_7087171 | IWGSC_CSS_4BS_scaff_4898025 | IWGSC_CSS_4DS_scaff_2283386 | Hecate_SCF:2677 |
| CINAU679 | IWGSC_CSS_4AL_scaff_7129586 | IWGSC_CSS_4BS_scaff_4955888 | IWGSC_CSS_4DS_scaff_2303219 | Hecate_CTG:136973104139252265 |
| CINAU680 | IWGSC_CSS_4AL_scaff_7105270 | IWGSC_CSS_4BS_scaff_4889586 | IWGSC_CSS_4DS_scaff_2296933 | Hecate_CTG:136973009649965651 |
| CINAU681 | IWGSC_CSS_4AL_scaff_7105270 | IWGSC_CSS_4BS_scaff_4921133 | IWGSC_CSS_4DS_scaff_2281062 | Hecate_CTG:136973907298127761 |
| CINAU682 | IWGSC_CSS_4AL_scaff_7105270 | IWGSC_CSS_4BS_scaff_4889586 | IWGSC_CSS_4DS_scaff_2296933 | Hecate_CTG:136973009649965651 |
| CINAU683 | IWGSC_CSS_4AL_scaff_7075662 | IWGSC_CSS_4BS_scaff_4960718 | IWGSC_CSS_4DS_scaff_1122851 | Hecate_SCF:1238 |
| CINAU684 | IWGSC_CSS_4AL_scaff_7106855 | IWGSC_CSS_4BS_scaff_4859213 | IWGSC_CSS_4DS_scaff_2316127 | Hecate_SCF:2444 |
| CINAU685 | IWGSC_CSS_4AL_scaff_639628 | IWGSC_CSS_4BS_scaff_4884331 | IWGSC_CSS_4DS_scaff_2321965 | Hecate_SCF:534 |
| CINAU686 | IWGSC_CSS_4AL_scaff_7173253 | IWGSC_CSS_4BS_scaff_4894873 | IWGSC_CSS_4DS_scaff_2324780 | Hecate_CTG:136973623830287219 |
| CINAU687 | IWGSC_CSS_4AL_scaff_7174192 | IWGSC_CSS_4BS_scaff_4898779 | IWGSC_CSS_4DS_scaff_2293539 | Hecate_CTG:136974268075349775 |
| CINAU688 | IWGSC_CSS_4AL_scaff_7173203 | IWGSC_CSS_4BS_scaff_4862292 | IWGSC_CSS_4DS_scaff_2295468 | Hecate_SCF:1822 |
| CINAU689 | IWGSC_CSS_4AL_scaff_7175720 | IWGSC_CSS_4BS_scaff_4962155 | IWGSC_CSS_4DS_scaff_2285924 | Hecate_CTG:136974199355879609 |
| CINAU690 | IWGSC_CSS_4AL_scaff_7136034 | IWGSC_CSS_4BS_scaff_4948517 | IWGSC_CSS_4DS_scaff_2325318 | Hecate_SCF:1437 |
| CINAU691 | IWGSC_CSS_4AL_scaff_7102651 | IWGSC_CSS_4BS_scaff_4890707 | IWGSC_CSS_4DS_scaff_1165433 | Hecate_SCF:5244 |
| CINAU692 | IWGSC_CSS_4AL_scaff_7136034 | IWGSC_CSS_4BS_scaff_4948517 | IWGSC_CSS_4DS_scaff_2325318 | Hecate_SCF:1506 |
| CINAU693 | IWGSC_CSS_4AL_scaff_7150296 | IWGSC_CSS_4BS_scaff_4883322 | IWGSC_CSS_4DS_scaff_2324298 | Hecate_SCF:266 |
| CINAU694 | IWGSC_CSS_4AL_scaff_7171600 | IWGSC_CSS_4BS_scaff_4963284 | IWGSC_CSS_4DS_scaff_2273651 | Hecate_SCF:528 |
| CINAU695 | IWGSC_CSS_4AL_scaff_7176618 | IWGSC_CSS_4BS_scaff_4941552 | IWGSC_CSS_4DS_scaff_2291894 | Hecate_CTG:136973271642987455 |
| CINAU696 | IWGSC_CSS_4AL_scaff_7153222 | IWGSC_CSS_4BS_scaff_4864606 | IWGSC_CSS_4DS_scaff_2301236 | Hecate_SCF:1649 |
| CINAU697 | IWGSC_CSS_4AL_scaff_7170184 | IWGSC_CSS_4BS_scaff_4929238 | IWGSC_CSS_4DS_scaff_2294610 | Hecate_SCF:1282 |
| CINAU698 | IWGSC_CSS_4AL_scaff_7135598 | IWGSC_CSS_4BS_scaff_4925255 | IWGSC_CSS_4DS_scaff_215326 | Hecate_CTG:136974478528755367 |
| CINAU699 | IWGSC_CSS_4AL_scaff_7136021 | IWGSC_CSS_4BS_scaff_4837767 | IWGSC_CSS_4DS_scaff_196370 | Hecate_SCF:19 |
| CINAU700 | IWGSC_CSS_4AL_scaff_7068307 | IWGSC_CSS_4BS_scaff_4962390 | IWGSC_CSS_4DS_scaff_1325877 | Hecate_CTG:136973756974258941 |
| CINAU701 | IWGSC_CSS_4AL_scaff_7176697 | IWGSC_CSS_4BS_scaff_4909391 | IWGSC_CSS_4DS_scaff_2300956 | Hecate_SCF:1510 |
| CINAU702 | IWGSC_CSS_4AL_scaff_7170407 | IWGSC_CSS_4BS_scaff_4935884 | IWGSC_CSS_4DS_scaff_2033184 | Hecate_SCF:1444 |
| CINAU703 | IWGSC_CSS_4AL_scaff_7085467 | IWGSC_CSS_4BS_scaff_4858956 | IWGSC_CSS_4DS_scaff_2302757 | Hecate_CTG:136973421966846659 |
| CINAU704 | IWGSC_CSS_4AL_scaff_7085467 | IWGSC_CSS_4BS_scaff_4902321 | IWGSC_CSS_4DS_scaff_2302757 | Hecate_SCF:346 |
| CINAU705 | IWGSC_CSS_4AL_scaff_7127998 | IWGSC_CSS_4BS_scaff_4901068 | IWGSC_CSS_4DS_scaff_2288468 | Hecate_SCF:2622 |
| CINAU706 | IWGSC_CSS_4AL_scaff_7106211 | IWGSC_CSS_4BS_scaff_4943331 | IWGSC_CSS_4DS_scaff_2292050 | Hecate_SCF:439 |
| CINAU707 | IWGSC_CSS_4AL_scaff_7124009 | IWGSC_CSS_4BS_scaff_4900325 | IWGSC_CSS_4DS_scaff_1807044 | Hecate_SCF:932 |
| CINAU708 | IWGSC_CSS_4AL_scaff_7123657 | IWGSC_CSS_4BS_scaff_4869572 | IWGSC_CSS_4DS_scaff_2326299 | Hecate_CTG:136973752679329279 |
| CINAU709 | IWGSC_CSS_4AL_scaff_7135598 | IWGSC_CSS_4BS_scaff_4938115 | IWGSC_CSS_4DS_scaff_2305086 | Hecate_CTG:136973310297651543 |
| CINAU710 | IWGSC_CSS_4AL_scaff_7140768 | IWGSC_CSS_4BS_scaff_4858663 | IWGSC_CSS_4DS_scaff_2287351 | Hecate_CTG:136972902275789079 |
| CINAU711 | IWGSC_CSS_4AL_scaff_7126999 | IWGSC_CSS_4BS_scaff_4909772 | IWGSC_CSS_4DS_scaff_2326689 | Hecate_CTG:136973911593098531 |
| CINAU712 | IWGSC_CSS_4AL_scaff_5518296 | IWGSC_CSS_4BS_scaff_2301182 | IWGSC_CSS_4DS_scaff_2293108 | Hecate_CTG:136971738339660641 |
| CINAU713 | IWGSC_CSS_4AL_scaff_7174822 | IWGSC_CSS_4BS_scaff_4932770 | IWGSC_CSS_4DS_scaff_2293108 | Hecate_SCF:803 |
| CINAU714 | IWGSC_CSS_4AL_scaff_7112332 | IWGSC_CSS_4BS_scaff_4946519 | IWGSC_CSS_4DS_scaff_2308145 | Hecate_SCF:465 |
| CINAU715 | IWGSC_CSS_4AL_scaff_7100634 | IWGSC_CSS_4BS_scaff_4947994 | IWGSC_CSS_4DS_scaff_2302719 | Hecate_SCF:1453 |
| CINAU716 | IWGSC_CSS_4AL_scaff_7032350 | IWGSC_CSS_4BS_scaff_4876643 | IWGSC_CSS_4DS_scaff_2289256 | Hecate_CTG:136974925205401393 |
| CINAU717 | IWGSC_CSS_4AL_scaff_7068037 | IWGSC_CSS_4BS_scaff_4872575 | IWGSC_CSS_4DS_scaff_2278293 | Hecate_SCF:1715 |
| CINAU718 | IWGSC_CSS_4AL_scaff_7162747 | IWGSC_CSS_4BS_scaff_4911608 | IWGSC_CSS_4DS_scaff_2316891 | Hecate_SCF:1249 |
| CINAU719 | IWGSC_CSS_4AL_scaff_7153141 | IWGSC_CSS_4BS_scaff_1315854 | IWGSC_CSS_4DS_scaff_2310006 | Hecate_SCF:1202 |
| CINAU720 | IWGSC_CSS_4AL_scaff_7098096 | IWGSC_CSS_4BS_scaff_4866598 | IWGSC_CSS_4DS_scaff_1591208 | Hecate_SCF:1703 |
| CINAU721 | IWGSC_CSS_4AL_scaff_1365428 | IWGSC_CSS_4BS_scaff_4957202 | IWGSC_CSS_4DS_scaff_2327045 | Hecate_CTG:136973714024622191 |
| CINAU722 | IWGSC_CSS_4AL_scaff_7127199 | IWGSC_CSS_4BS_scaff_4866863 | IWGSC_CSS_4DS_scaff_2290541 | Hecate_CTG:136972021807509397 |
| CINAU723 | IWGSC_CSS_4AL_scaff_7143233 | IWGSC_CSS_4BS_scaff_2560542 | IWGSC_CSS_4DS_scaff_2320488 | Hecate_SCF:26680 |
| CINAU724 | IWGSC_CSS_4AL_scaff_7105406 | IWGSC_CSS_4BS_scaff_4957913 | IWGSC_CSS_4DS_scaff_86561 | Hecate_SCF:200 |
| CINAU725 | IWGSC_CSS_4AL_scaff_7098212 | IWGSC_CSS_4BS_scaff_4887494 | IWGSC_CSS_4DS_scaff_2324379 | Hecate_SCF:372 |
| CINAU726 | IWGSC_CSS_4AL_scaff_7120959 | IWGSC_CSS_4BS_scaff_4899342 | IWGSC_CSS_4DS_scaff_2307013 | Hecate_CTG:136975097004070519 |
| CINAU727 | IWGSC_CSS_4AL_scaff_7032976 | IWGSC_CSS_4BS_scaff_908529 | IWGSC_CSS_4DS_scaff_2303375 | Hecate_SCF:1995 |
| CINAU728 | IWGSC_CSS_4AL_scaff_7062782 | IWGSC_CSS_4BS_scaff_114263 | IWGSC_CSS_4DS_scaff_2293547 | Hecate_SCF:1615 |
| CINAU729 | IWGSC_CSS_4AL_scaff_7153760 | IWGSC_CSS_4BS_scaff_4942770 | IWGSC_CSS_4DS_scaff_2306670 | Hecate_CTG:136975221558124357 |
| CINAU730 | IWGSC_CSS_4AL_scaff_7073282 | IWGSC_CSS_4BS_scaff_4958074 | IWGSC_CSS_4DS_scaff_1510512 | Hecate_SCF:827 |
| CINAU731 | IWGSC_CSS_4AL_scaff_7088291 | IWGSC_CSS_4BS_scaff_4913081 | IWGSC_CSS_4DS_scaff_898911 | Hecate_SCF:2960 |
| CINAU732 | IWGSC_CSS_4AL_scaff_7085322 | IWGSC_CSS_4BS_scaff_4930624 | IWGSC_CSS_4DS_scaff_2292443 | Hecate_CTG:136974843600996697 |
| CINAU733 | IWGSC_CSS_4AL_scaff_7103707 | IWGSC_CSS_4BS_scaff_4898487 | IWGSC_CSS_4DS_scaff_2292443 | Hecate_SCF:909 |
| CINAU734 | IWGSC_CSS_4AL_scaff_7176085 | IWGSC_CSS_4BS_scaff_4912900 | IWGSC_CSS_4DS_scaff_2283662 | Hecate_SCF:3426 |
| CINAU735 | IWGSC_CSS_4AL_scaff_7093114 | IWGSC_CSS_4BS_scaff_4873209 | IWGSC_CSS_4DS_scaff_2278641 | Hecate_CTG:136974259485440371 |
| CINAU736 | IWGSC_CSS_4AL_scaff_7170849 | IWGSC_CSS_4BS_scaff_4949839 | IWGSC_CSS_4DS_scaff_2302220 | Hecate_CTG:136974392629440871 |
| CINAU737 | IWGSC_CSS_4AL_scaff_7040796 | IWGSC_CSS_4BS_scaff_4869572 | IWGSC_CSS_4DS_scaff_2316979 | Hecate_SCF:732 |
| CINAU738 | IWGSC_CSS_4AL_scaff_2510301 | IWGSC_CSS_4BS_scaff_4860506 | IWGSC_CSS_4DS_scaff_1654576 | Hecate_CTG:136974538658310045 |
| CINAU739 | IWGSC_CSS_4AL_scaff_7083673 | IWGSC_CSS_4BS_scaff_4863914 | IWGSC_CSS_4DS_scaff_1920120 | Hecate_SCF:4746 |
| CINAU740 | IWGSC_CSS_4AL_scaff_2619375 | IWGSC_CSS_4BS_scaff_4863914 | IWGSC_CSS_4DS_scaff_1920120 | Hecate_SCF:893 |
| CINAU741 | IWGSC_CSS_4AL_scaff_7155047 | IWGSC_CSS_4BS_scaff_4959594 | IWGSC_CSS_4DS_scaff_2284808 | Hecate_CTG:136972425534399159 |
| CINAU742 | IWGSC_CSS_4AS_scaff_6003230 | IWGSC_CSS_4BS_scaff_4872759 | IWGSC_CSS_4DS_scaff_612669 | Hecate_SCF:781 |
| CINAU743 | IWGSC_CSS_4AL_scaff_7134002 | IWGSC_CSS_4BS_scaff_4862233 | IWGSC_CSS_4DS_scaff_2325707 | Hecate_CTG:136975032579564567 |
| CINAU744 | IWGSC_CSS_4AL_scaff_7022558 | IWGSC_CSS_4BS_scaff_4907691 | IWGSC_CSS_4DS_scaff_2315421 | Hecate_SCF:200 |
| CINAU745 | IWGSC_CSS_4AL_scaff_7118203 | IWGSC_CSS_4BS_scaff_4879838 | IWGSC_CSS_4DS_scaff_2277587 | Hecate_SCF:2671 |
| CINAU746 | IWGSC_CSS_4AL_scaff_7166106 | IWGSC_CSS_4BS_scaff_4882269 | IWGSC_CSS_4DS_scaff_2293441 | Hecate_CTG:136975006809763697 |
| CINAU747 | IWGSC_CSS_4AL_scaff_7103619 | IWGSC_CSS_4BS_scaff_4918399 | IWGSC_CSS_4DS_scaff_367837 | Hecate_SCF:200 |
| CINAU748 | IWGSC_CSS_4AL_scaff_7085362 | IWGSC_CSS_4BS_scaff_4867673 | IWGSC_CSS_4DS_scaff_2325901 | Hecate_SCF:1635 |
| CINAU749 | IWGSC_CSS_4AL_scaff_7172321 | IWGSC_CSS_4BS_scaff_4952865 | IWGSC_CSS_4DS_scaff_1477646 | Hecate_SCF:1656 |
| CINAU750 | IWGSC_CSS_4AL_scaff_7072073 | IWGSC_CSS_4BS_scaff_4944090 | IWGSC_CSS_4DS_scaff_2317565 | Hecate_SCF:3140 |
| CINAU751 | IWGSC_CSS_4AL_scaff_7063359 | IWGSC_CSS_4BS_scaff_4956735 | IWGSC_CSS_4DS_scaff_2308818 | Hecate_SCF:2150 |
| CINAU752 | IWGSC_CSS_4AL_scaff_5800627 | IWGSC_CSS_4BS_scaff_4956735 | IWGSC_CSS_4DS_scaff_2308818 | Hecate_SCF:1365 |
| CINAU753 | IWGSC_CSS_4AL_scaff_7141603 | IWGSC_CSS_4BS_scaff_4891756 | IWGSC_CSS_4DS_scaff_2306888 | Hecate_SCF:351 |
| CINAU754 | IWGSC_CSS_4AL_scaff_7140405 | IWGSC_CSS_4BS_scaff_4944033 | IWGSC_CSS_4DS_scaff_2294583 | Hecate_SCF:2847 |
| CINAU755 | IWGSC_CSS_4AL_scaff_7154030 | IWGSC_CSS_4BS_scaff_4868257 | IWGSC_CSS_4DS_scaff_2312263 | Hecate_SCF:528 |
| CINAU756 | IWGSC_CSS_4AL_scaff_7111247 | IWGSC_CSS_4BS_scaff_4961277 | IWGSC_CSS_4DS_scaff_2273747 | Hecate_SCF:1187 |
| CINAU757 | IWGSC_CSS_4AL_scaff_7094098 | IWGSC_CSS_4BS_scaff_4882386 | IWGSC_CSS_4DS_scaff_2300056 | Hecate_CTG:136974134931378901 |
| CINAU758 | IWGSC_CSS_4AL_scaff_7162989 | IWGSC_CSS_4BS_scaff_4871207 | IWGSC_CSS_4DS_scaff_2315275 | Hecate_SCF:301 |
| CINAU759 | IWGSC_CSS_4AL_scaff_7163632 | IWGSC_CSS_4BS_scaff_4863900 | IWGSC_CSS_4DS_scaff_2296642 | Hecate_CTG:136974452758980233 |
| CINAU760 | IWGSC_CSS_4AS_scaff_5955042 | IWGSC_CSS_4BS_scaff_4959908 | IWGSC_CSS_4DS_scaff_2301338 | Hecate_SCF:3292 |
| CINAU761 | IWGSC_CSS_4AL_scaff_4377497 | IWGSC_CSS_4BS_scaff_4959908 | IWGSC_CSS_4DS_scaff_2301338 | Hecate_SCF:3292 |
| CINAU762 | IWGSC_CSS_4AL_scaff_7070388 | IWGSC_CSS_4BS_scaff_4942770 | IWGSC_CSS_4DS_scaff_2316545 | Hecate_SCF:334 |
| CINAU763 | IWGSC_CSS_4AL_scaff_7070388 | IWGSC_CSS_4BS_scaff_4942770 | IWGSC_CSS_4DS_scaff_2316545 | Hecate_SCF:334 |
| CINAU764 | IWGSC_CSS_4AL_scaff_4694471 | IWGSC_CSS_4BS_scaff_4924357 | IWGSC_CSS_4DS_scaff_2304227 | Hecate_CTG:136973516456099221 |
| CINAU765 | IWGSC_CSS_4AL_scaff_7106463 | IWGSC_CSS_4BS_scaff_4945241 | IWGSC_CSS_4DS_scaff_2307391 | Hecate_SCF:190 |
| CINAU766 | IWGSC_CSS_4AL_scaff_7097070 | IWGSC_CSS_4BS_scaff_4934795 | IWGSC_CSS_4DS_scaff_2323618 | Hecate_SCF:1409 |
| CINAU767 | IWGSC_CSS_4AL_scaff_7102499 | IWGSC_CSS_4BS_scaff_787157 | IWGSC_CSS_4DS_scaff_2284832 | Hecate_SCF:632 |
| CINAU768 | IWGSC_CSS_4AL_scaff_7143472 | IWGSC_CSS_4BS_scaff_4877758 | IWGSC_CSS_4DS_scaff_2284832 | Hecate_SCF:967 |
| CINAU769 | IWGSC_CSS_4AL_scaff_61032 | IWGSC_CSS_4BS_scaff_2423753 | IWGSC_CSS_4DS_scaff_2297593 | Hecate_SCF:845 |
| CINAU770 | IWGSC_CSS_4AL_scaff_7130019 | IWGSC_CSS_4BS_scaff_4888528 | IWGSC_CSS_4DS_scaff_2325871 | Hecate_SCF:1995 |
| CINAU771 | IWGSC_CSS_4AL_scaff_7133975 | IWGSC_CSS_4BS_scaff_4944154 | IWGSC_CSS_4DS_scaff_1302219 | Hecate_CTG:136975152838665691 |
| CINAU772 | IWGSC_CSS_4AL_scaff_7102341 | IWGSC_CSS_4BS_scaff_4957317 | IWGSC_CSS_4DS_scaff_2291774 | Hecate_SCF:498 |
| CINAU773 | IWGSC_CSS_4AL_scaff_7152861 | IWGSC_CSS_4BS_scaff_4937582 | IWGSC_CSS_4DS_scaff_2325639 | Hecate_CTG:136974886550674799 |
| CINAU774 | IWGSC_CSS_4AL_scaff_53735 | IWGSC_CSS_4BS_scaff_4924087 | IWGSC_CSS_4DS_scaff_2290638 | Hecate_SCF:1421 |
| CINAU775 | IWGSC_CSS_4AL_scaff_5677530 | IWGSC_CSS_4BS_scaff_4924087 | IWGSC_CSS_4DS_scaff_2290638 | Hecate_SCF:942 |
| CINAU776 | IWGSC_CSS_4AL_scaff_7170553 | IWGSC_CSS_4BS_scaff_4916306 | IWGSC_CSS_4DS_scaff_2290405 | Hecate_SCF:656 |
| CINAU777 | IWGSC_CSS_4AL_scaff_7094315 | IWGSC_CSS_4BS_scaff_4916572 | IWGSC_CSS_4DS_scaff_2290960 | Hecate_SCF:3326 |
| CINAU778 | IWGSC_CSS_4AL_scaff_7125202 | IWGSC_CSS_4BS_scaff_4862584 | IWGSC_CSS_4DS_scaff_2311530 | Hecate_CTG:136974736226823039 |
| CINAU779 | IWGSC_CSS_4AL_scaff_7156080 | IWGSC_CSS_4BS_scaff_4915475 | IWGSC_CSS_4DS_scaff_2275695 | Hecate_SCF:20 |
| CINAU780 | IWGSC_CSS_4AL_scaff_7171949 | IWGSC_CSS_4BS_scaff_4915475 | IWGSC_CSS_4DS_scaff_2275695 | Hecate_SCF:2 |
| CINAU781 | IWGSC_CSS_4AL_scaff_7086760 | IWGSC_CSS_4BS_scaff_4889626 | IWGSC_CSS_4DS_scaff_2295389 | Hecate_SCF:1618 |
| CINAU782 | IWGSC_CSS_4AL_scaff_7122108 | IWGSC_CSS_4BS_scaff_4963232 | IWGSC_CSS_4DS_scaff_2276661 | Hecate_SCF:3358 |
| CINAU783 | IWGSC_CSS_4AL_scaff_7132733 | IWGSC_CSS_4BS_scaff_4899363 | IWGSC_CSS_4DS_scaff_2300859 | Hecate_SCF:985 |
| CINAU784 | IWGSC_CSS_4AL_scaff_7063462 | IWGSC_CSS_4BS_scaff_4869333 | IWGSC_CSS_4DS_scaff_108716 | Hecate_SCF:3351 |
| CINAU785 | IWGSC_CSS_4AL_scaff_7078006 | IWGSC_CSS_4BS_scaff_4884039 | IWGSC_CSS_4DS_scaff_2290317 | Hecate_SCF:118 |
| CINAU786 | IWGSC_CSS_4AL_scaff_7156419 | IWGSC_CSS_4BS_scaff_4868409 | IWGSC_CSS_4DS_scaff_2317536 | Hecate_CTG:136972588743189791 |
| CINAU787 | IWGSC_CSS_4AL_scaff_7127028 | IWGSC_CSS_4BS_scaff_4919222 | IWGSC_CSS_4DS_scaff_2275952 | Hecate_SCF:2655 |
| CINAU788 | IWGSC_CSS_4AL_scaff_7162989 | IWGSC_CSS_4BS_scaff_2307753 | IWGSC_CSS_4DS_scaff_2301779 | Hecate_CTG:136972112001821043 |
| CINAU789 | IWGSC_CSS_4AL_scaff_7169510 | IWGSC_CSS_4BS_scaff_4870164 | IWGSC_CSS_4DS_scaff_2305716 | Hecate_SCF:120 |
| CINAU790 | IWGSC_CSS_4AL_scaff_7143734 | IWGSC_CSS_4BS_scaff_4962439 | IWGSC_CSS_4DS_scaff_2321766 | Hecate_CTG:136973353247328549 |
| CINAU791 | IWGSC_CSS_4AL_scaff_7018363 | IWGSC_CSS_4BS_scaff_4917102 | IWGSC_CSS_4DS_scaff_2297513 | Hecate_CTG:136974139226365975 |
| CINAU792 | IWGSC_CSS_4AL_scaff_7127651 | IWGSC_CSS_4BS_scaff_4937384 | IWGSC_CSS_4DS_scaff_2297250 | Hecate_SCF:226 |
| CINAU793 | IWGSC_CSS_4AL_scaff_7145539 | IWGSC_CSS_4BS_scaff_4935784 | IWGSC_CSS_4DS_scaff_2304434 | Hecate_SCF:612 |
| CINAU794 | IWGSC_CSS_4AL_scaff_7127028 | IWGSC_CSS_4BS_scaff_4919222 | IWGSC_CSS_4DS_scaff_2324973 | Hecate_CTG:136973155678862285 |
| CINAU795 | IWGSC_CSS_4AL_scaff_7163575 | IWGSC_CSS_4BS_scaff_4916819 | IWGSC_CSS_4DS_scaff_2283971 | Hecate_SCF:754 |
| CINAU796 | IWGSC_CSS_4AL_scaff_379239 | IWGSC_CSS_4BS_scaff_4942385 | IWGSC_CSS_4DS_scaff_2286567 | Hecate_SCF:3907 |
| CINAU797 | IWGSC_CSS_4AL_scaff_7038004 | IWGSC_CSS_4BS_scaff_4939931 | IWGSC_CSS_4DS_scaff_2289313 | Hecate_SCF:930 |
| CINAU798 | IWGSC_CSS_4AL_scaff_7116319 | IWGSC_CSS_4BS_scaff_4944381 | IWGSC_CSS_4DS_scaff_2274964 | Hecate_SCF:1220 |
| CINAU799 | IWGSC_CSS_4AL_scaff_7100611 | IWGSC_CSS_4BS_scaff_4902999 | IWGSC_CSS_4DS_scaff_2289799 | Hecate_SCF:5842 |
| CINAU800 | IWGSC_CSS_4AL_scaff_7092533 | IWGSC_CSS_4BS_scaff_4927251 | IWGSC_CSS_4DS_scaff_2320147 | Hecate_SCF:1780 |
| CINAU801 | IWGSC_CSS_4AL_scaff_897968 | IWGSC_CSS_4BS_scaff_4885384 | IWGSC_CSS_4DS_scaff_2316006 | Hecate_SCF:4006 |
| CINAU802 | IWGSC_CSS_4AL_scaff_7169510 | IWGSC_CSS_4BS_scaff_4870164 | IWGSC_CSS_4DS_scaff_2305716 | Hecate_SCF:4291 |
| CINAU803 | IWGSC_CSS_4AL_scaff_7112880 | IWGSC_CSS_4BS_scaff_4910097 | IWGSC_CSS_4DS_scaff_2303143 | Hecate_SCF:3038 |
| CINAU804 | IWGSC_CSS_4AL_scaff_7125421 | IWGSC_CSS_4BS_scaff_4875486 | IWGSC_CSS_4DS_scaff_2288657 | Hecate_SCF:132 |
| CINAU805 | IWGSC_CSS_4AL_scaff_6933721 | IWGSC_CSS_4BS_scaff_4935305 | IWGSC_CSS_4DS_scaff_523211 | Hecate_SCF:55 |
| CINAU806 | IWGSC_CSS_4AL_scaff_7109322 | IWGSC_CSS_4BS_scaff_4888726 | IWGSC_CSS_4DS_scaff_2326315 | Hecate_SCF:903 |
| CINAU807 | IWGSC_CSS_4AL_scaff_7123718 | IWGSC_CSS_4BS_scaff_4895311 | IWGSC_CSS_4DS_scaff_2309246 | Hecate_SCF:960 |
| CINAU808 | IWGSC_CSS_4AL_scaff_7096467 | IWGSC_CSS_4BS_scaff_4909223 | IWGSC_CSS_4DS_scaff_2295069 | Hecate_CTG:136974929500363285 |
| CINAU809 | IWGSC_CSS_4AL_scaff_7173114 | IWGSC_CSS_4BS_scaff_4863544 | IWGSC_CSS_4DS_scaff_2321923 | Hecate_SCF:1696 |
| CINAU810 | IWGSC_CSS_4AL_scaff_7127028 | IWGSC_CSS_4BS_scaff_4919222 | IWGSC_CSS_4DS_scaff_2324973 | Hecate_CTG:136973155678862285 |
| CINAU811 | IWGSC_CSS_4AL_scaff_7079223 | IWGSC_CSS_4BS_scaff_4866908 | IWGSC_CSS_4DS_scaff_2282728 | Hecate_SCF:1225 |
| CINAU812 | IWGSC_CSS_4AL_scaff_7106921 | IWGSC_CSS_4BS_scaff_4875486 | IWGSC_CSS_4DS_scaff_2325367 | Hecate_SCF:264 |
| CINAU813 | IWGSC_CSS_4AL_scaff_7154078 | IWGSC_CSS_4BS_scaff_4963109 | IWGSC_CSS_4DS_scaff_2270703 | Hecate_SCF:1385 |
| CINAU814 | IWGSC_CSS_4AL_scaff_7123664 | IWGSC_CSS_4BS_scaff_4899773 | IWGSC_CSS_4DS_scaff_2311135 | Hecate_SCF:1212 |
| CINAU815 | IWGSC_CSS_4AL_scaff_7067815 | IWGSC_CSS_4BS_scaff_4898810 | IWGSC_CSS_4DS_scaff_2300859 | Hecate_SCF:836 |
| CINAU816 | IWGSC_CSS_4AL_scaff_7173726 | IWGSC_CSS_4BS_scaff_4961544 | IWGSC_CSS_4DS_scaff_2301861 | Hecate_SCF:865 |
| CINAU817 | IWGSC_CSS_4AL_scaff_7063488 | IWGSC_CSS_4BS_scaff_4879560 | IWGSC_CSS_4DS_scaff_2319488 | Hecate_CTG:136974547248253315 |
| CINAU818 | IWGSC_CSS_4AL_scaff_7176420 | IWGSC_CSS_4BS_scaff_4951238 | IWGSC_CSS_4DS_scaff_2297951 | Hecate_SCF:276 |
| CINAU819 | IWGSC_CSS_4AL_scaff_7068621 | IWGSC_CSS_4BS_scaff_4861114 | IWGSC_CSS_4DS_scaff_2288122 | Hecate_CTG:136974835011058407 |
| CINAU820 | IWGSC_CSS_4AL_scaff_7143904 | IWGSC_CSS_4BS_scaff_4903805 | IWGSC_CSS_4DS_scaff_2308821 | Hecate_SCF:415 |
| CINAU821 | IWGSC_CSS_4AL_scaff_7126034 | IWGSC_CSS_4BS_scaff_3250029 | IWGSC_CSS_4DS_scaff_2274560 | Hecate_CTG:136973653895049189 |
| CINAU822 | IWGSC_CSS_4AL_scaff_7090235 | IWGSC_CSS_4BS_scaff_4928731 | IWGSC_CSS_4DS_scaff_1975210 | Hecate_SCF:695 |
| CINAU823 | IWGSC_CSS_4AL_scaff_7084131 | IWGSC_CSS_4BS_scaff_4875486 | IWGSC_CSS_4DS_scaff_2325367 | Hecate_SCF:263 |
| CINAU824 | IWGSC_CSS_4AL_scaff_4694471 | IWGSC_CSS_4BS_scaff_4924357 | IWGSC_CSS_4DS_scaff_2292909 | Hecate_CTG:136973967427641017 |
| CINAU825 | IWGSC_CSS_4AL_scaff_7151770 | IWGSC_CSS_4BS_scaff_4865990 | IWGSC_CSS_4DS_scaff_2311417 | Hecate_SCF:2248 |
| CINAU826 | IWGSC_CSS_4AL_scaff_7023082 | IWGSC_CSS_4BS_scaff_4885384 | IWGSC_CSS_4DS_scaff_2316006 | Hecate_SCF:4006 |
| CINAU827 | IWGSC_CSS_4AL_scaff_7085010 | IWGSC_CSS_4BS_scaff_4901723 | IWGSC_CSS_4DS_scaff_2322330 | Hecate_CTG:136972073347061407 |
| CINAU828 | IWGSC_CSS_4AL_scaff_7174903 | IWGSC_CSS_4BS_scaff_4887549 | IWGSC_CSS_4DS_scaff_2320985 | Hecate_SCF:782 |
| CINAU829 | IWGSC_CSS_4AL_scaff_7139664 | IWGSC_CSS_4BS_scaff_4867424 | IWGSC_CSS_4DS_scaff_1516782 | Hecate_SCF:1496 |
| CINAU830 | IWGSC_CSS_4AL_scaff_7012377 | IWGSC_CSS_4BS_scaff_4936645 | IWGSC_CSS_4DS_scaff_2289799 | Hecate_CTG:136972416944460909 |
| CINAU831 | IWGSC_CSS_4AL_scaff_7085282 | IWGSC_CSS_4BS_scaff_4863679 | IWGSC_CSS_4DS_scaff_2284288 | Hecate_CTG:136975036874538879 |
| CINAU832 | IWGSC_CSS_4AL_scaff_7071864 | IWGSC_CSS_4BS_scaff_4913526 | IWGSC_CSS_4DS_scaff_2320231 | Hecate_SCF:4783 |
| CINAU833 | IWGSC_CSS_4AL_scaff_7085010 | IWGSC_CSS_4BS_scaff_4901723 | IWGSC_CSS_4DS_scaff_2322330 | Hecate_CTG:136973095549307307 |
| CINAU834 | IWGSC_CSS_4AL_scaff_7162989 | IWGSC_CSS_4BS_scaff_2307753 | IWGSC_CSS_4DS_scaff_2301779 | Hecate_CTG:136972112001821043 |
| CINAU835 | IWGSC_CSS_4AL_scaff_7134133 | IWGSC_CSS_4BS_scaff_4897750 | IWGSC_CSS_4DS_scaff_2321534 | Hecate_SCF:1590 |
