# Supplementary material for: Development of intron targeting (IT) markers specific for chromosome arm 4VS of Haynaldia villosa by chromosome sorting and next-generation sequencing
Source: BMC Genomics. 2017 Feb 15;18:167. doi: 10.1186/s12864-017-3567-z (PMC5310052; doi:10.1186/s12864-017-3567-z)
Supplement: Additional file 5: Table S2. — The sequences of forward and reverse primers and the size of intron of the IT markers (DOCX 48 kb) [file 12864_2017_3567_MOESM5_ESM.docx]

| Marker no.  Table S2 The sequences of forward and reverse primers and the size of intron of the IT markers | Forward primer (5′→3′) | Reverse primer (5′→3′) | Intron size of A genome | Intron size of B genome | Intron size of D genome | Intron size of V genome | Type |
| --- | --- | --- | --- | --- | --- | --- | --- |
| CINAU604 | TTGTATGATGCTGCGACGAC | TCTCCTTCTGCACAGAGCAA | 145 | 189 | 191 | 158 | Type VI |
| CINAU605 | AGCTCGCCATCATCTCCA | ACAGGTATGCCTTGCTGAGG | 584 | 614 | 566 | 869 | Type VI |
| CINAU606 | TCATATGATGCAAGTTCTTGAGG | TCTCCTTCTGCCAGTCATCC | 607 | 553 | 605 | 1352 | Type VI |
| CINAU607 | GAACGCCATCGTCATCAAC | CTTGTACCGGCCATTGCT | 196 | 258 | 196 | 174 | Type VI |
| CINAU608 | CCACCTGAAGCAAGACAAAA | GAACCAGGTCGACTAATTCAGG | 441 | 517 | 337 | 403 | Type VI |
| CINAU609 | CCCAGATCGACCTCTTCCAG | CACCACCGTGGGGATGTA | 106 | 83 | 95 | 80 | Type VI |
| CINAU610 | ACCCTAAAGCCCAGAGGAAG | CCCATGGACTTGGTGAACA | 126 | 131 | 118 | 102 | Type VI |
| CINAU611 | TGCTGGCGAGGGTATTATCA | AGCATCACCCCGGATGTTAA | 397 | 415 | 417 | 431 | Type II |
| CINAU612 | CTTGCCCGTGATGCCATTC | GCTGCCAATCCTCAATTTGC | 619 | 623 | 620 | 587 | Type II |
| CINAU613 | TCGCCAATGCCTTCTATCCT | TCGCATCGGTAGACACATCA | 153 | 152 | 91 | 142 | Type I |
| CINAU614 | AGCCTAATCATACCACGGTCA | GCGCAGTTCTGGTTTCTGTT | 213 | 198 | 201 | 189 | Type V |
| CINAU615 | GCAGTTCCGGTTTCTGTTCT | TGAGCCTAATCATACCACGGT | 213 | 198 | 201 | 189 | Type V |
| CINAU616 | TGCTGGTTGACAATTTCCACA | GCTTGGCTTCAGAAGGGAA | 1224 | 560 | 1147 | 479 | Type II |
| CINAU617 | GCTTCCCTGCATCCTCATCA | GTCCTCAGCCTCATCCTCTT | 190 | 190 | 187 | 175 | Type IV |
| CINAU618 | CCTCAGACTGGATGCACTTG | CGCATCGATTTCACTGGCAT | 374 | 665 | 422 | 475 | Type I |
| CINAU619 | CGTGTCGGTGAACCTCTTT | GTTCCAAGACCTCCACATGG | 773 | 2334 | 924 | 894 | Type IV |
| CINAU620 | TCTCTTCTCTTGACGCAGCG | CTCCGTGCTCAGGTAGTCAT | 74 | 75 | 74 | 109 | Type VI |
| CINAU621 | CACCTTCCCTATCTCGCTCA | ACGTCTGAGTAGTCCGGTTT | 573 | 555 | 595 | 533 | Type VI |
| CINAU622 | CCAAGAGGCGGATGGTATCA | CCGGCCATGTTGCATTTATG | 349 | 349 | 349 | 306 | Type VI |
| CINAU623 | AAGCACTACAACTGGAAGCC | GATCTCGCTAAAAGTGCGGG | 516 | 512 | 625 | 419 | Type V |
| CINAU624 | CCATCTTCACGTTTGCCTGT | ACCTGTTGATCAAGTGGCAA | 0 | 0 | 512 | 476 | Type V |
| CINAU625 | CGCTTGCAATTGTGGATGAAC | TGAGGCTTCAATTTCCTGATCA | 215 | 212 | 217 | 135 | Type V |
| CINAU626 | GGAAGAACCCTGCGAATGAA | CTGCCCAGACAGTGAGGAAG | 195 | 195 | 195 | 162 | Type VI |
| CINAU627 | GGACAAGCTGTGGGACGA | CACCTCCACCAACATCCATG | 118 | 118 | 108 | 130 | Type V |
| CINAU628 | ATCATCTCTACTCGCAGCCC | CGGAGAGGACCTGATTCTGG | 149 | 152 | 151 | 255 | Type VI |
| CINAU629 | AGAACCACCAACAATCAGCTG | AGTTGTTCGAGCATTCACCG | 472 | 316 | 316 | 539 | Type IV |
| CINAU630 | GAGGGAAACTGTGCACGATG | TTCTGTGCCATGACGATTGT | 274 | 259 | 276 | 244 | Type II |
| CINAU631 | CAAGTACTCCTCTACGTCGTC | CCCCAAAAGAAAGAAGACTGGA | 222 | 204 | 205 | 252 | Type VI |
| CINAU632 | GGGTTTGGGAGCGCAAATAA | ACCAACCCCTTTCTTCCAGG | 101 | 134 | 80 | 151 | Type I |
| CINAU633 | CGCAAAGTTCTATCTCCGCC | AGTGTAGTAAGCTCTTCCATCGA | 386 | 784 | 685 | 482 | Type II |
| CINAU634 | ACCAGCTATTTATTACATGCCGG | GGGTTAGCTCTGTTCTGCGT | 938 | 934 | 1289 | 878 | Type II |
| CINAU635 | GGATCTACCCCATGTATTCACC | TCCTTCACTGTCCAGCAAGA | 270 | 268 | 199 | 249 | Type II |
| CINAU636 | ACCTTTGCGTGAAGTTCGTT | ACATCATGTAATCTGCGGCA | 665 | 634 | 640 | 617 | Type II |
| CINAU637 | TTTGGATTTAATGGCGGCGC | TTGTTGTTGGTGGTGAGTCTG | 306 | 404 | 353 | 485 | Type II |
| CINAU638 | CAATCTGCGGGGTTCCATC | ACTCACCAGTACAGAATCATCG | 363 | 295 | 298 | 393 | Type III |
| CINAU639 | CTGGTGACGGCGCTGAAC | CCGTCATGATCGCTCTCCT | 107 | 97 | 104 | 73 | Type III |
| CINAU640 | TGTTGGTGCTCAGAAAGTGA | TCCTAACAAGGGCCAACGC | 347 | 365 | 347 | 387 | Type II |
| CINAU641 | GGAAGTGGGTGCAGGAGAG | ATGGTCGGCGAACTGGTG | 1231 | 1558 | 1266 | 120 | Type I |
| CINAU642 | AGGGATTGTGATAGCAAGGC | AGCTCTCCTCACTTTCTTCCT | 1571 | 1582 | 1541 | 833 | Type IV |
| CINAU643 | ATGAAGTCCATCGTGCCCG | GGATCATACCCACGAACAGC | 965 | 1048 | 932 | 906 | Type I |
| CINAU644 | ACCATGGAACTGCAGAAGAA | GTCTTTTCTCAGCTTCCCGG | 81 | 77 | 81 | 167 | TypeVI |
| CINAU645 | GGAGCTTATGACACCGAAGAG | ATGGCCACGTACTCCTCTCT | 118 | 118 | 116 | 131 | TypeVI |
| CINAU646 | CATCGGTACTACGGGCGAT | TGCGGGTACTTCATCCTCAT | 298 | 304 | 307 | 445 | TypeVI |
| CINAU647 | TTGGTGGTCTCTGTACGCTT | CCCAGAATGCGGCTTTCATAA | 271 | 697 | 269 | 185 | TypeVI |
| CINAU648 | AGCCTCTCCTCCCTCAATCT | TGCTTCCACCTCAAATTGAACAT | 269 | 308 | 281 | 233 | Type II |
| CINAU649 | TTCAGAAACTTGGCTTTCCA | ACATCACAGGAGCCAACGAT | 91 | 88 | 101 | 72 | Type V |
| CINAU650 | GGAACGAAGGAGGTGAGAGG | CTGCCACTCCACCTTCTTCT | 82 | 94 | 108 | 119 | Type VI |
| CINAU651 | AGTGATTTGTGGGTAACTGCAA | GGATGACGTGGATGTGGACA | 335 | 453 | 325 | 389 | Type VI |
| CINAU652 | GTCGAATCAGTGCTTCAATCCA | TAAGTTTGGGGCAGTTTGTGG | 95 | 452 | 595 | 173 | Type V |
| CINAU653 | ACAAGATCATGCTCGCCAAG | CCTCGCGTTGATGAACCAG | 120 | 115 | 119 | 139 | Type IV |
| CINAU654 | GCGTGCTACAATTAGGCTCT | ATGCAGCCATACTTGATCGC | 563 | 563 | 726 | 374 | Type II |
| CINAU655 | GAGCACCACCCAAAGCATATT | AGTTCCTCAGCAGAAACGTG | 273 | 271 | 269 | 534 | Type I |
| CINAU656 | ACTCATGGATGGTTCTCGGA | GCACTCAACAATCCTCCCAG | 90 | 598 | 91 | 460 | Type VI |
| CINAU657 | CACAAGAGCCAGGGGAACA | GATGTTCTCGTGCGCCATCC | 103 | 106 | 95 | 135 | Type VI |
| CINAU658 | AGTCTTTGCTGCCATTGTTCT | GGCCTCCAAAGCTTCAACAC | 764 | 526 | 532 | 507 | Type VI |
| CINAU659 | GCGAGTCGGTCATGCTCAT | CTTGTACTTGGCGACCTCCT | 217 | 218 | 213 | 229 | Type V |
| CINAU660 | GGATGAGATTGGCCGTAATG | CAGCCTCTGTCCTGGAGATG | 374 | 401 | 374 | 321 | Type VI |
| CINAU661 | CACTTGCATACCTGAAGATGCT | CATGATCCATTGTGGCGTTA | 399 | 352 | 394 | 378 | Type V |
| CINAU662 | GCTTCCGAGTAGAATTTGACG | GGCCCTCCTCAGATTTTTCT | 147 | 133 | 136 | 88 | Type IV |
| CINAU663 | GCAGACCTCCAAGAAGCAG | GATGCCTCTTGAACGACTCC | 727 | 727 | 651 | 612 | Type IV |
| CINAU664 | TCTCCTCAATGGTTGCCTCT | GGCATCAACACTGCCATCTT | 497 | 495 | 497 | 597 | Type VI |
| CINAU665 | GCTCGGATGCAATTATTGTTGA | ATGGTCCTTCGCAGCTGTTA | 639 | 575 | 576 | 508 | Type IV |
| CINAU666 | GCTCAAGGACAACGGGAAC | CACCCATGTCCTCCAATTTCT | 379 | 330 | 310 | 403 | Type I |
| CINAU667 | CCTGGTCCAATCCTTTGTTCA | TGTTGAGAAGACTATGATGCGT | 329 | 361 | 361 | 344 | Type II |
| CINAU668 | AGAGGTGTATGCCGTGTGAA | GGAGAAACATCACCACGCAA | 402 | 401 | 401 | 387 | Type VI |
| CINAU669 | AGCTTCATTTGTAGGCTGTGC | AGAGGTGTATGCCGTGTGAA | 402 | 401 | 401 | 387 | Type VI |
| CINAU670 | CCAAGTACGAGATGGGAGGG | GTCCAGCTCGATGAAGTCCT | 103 | 78 | 97 | 113 | Type VI |
| CINAU671 | GGAGCTCTACTGCCTCGAG | CTTCCCAGCACCAGTCTCA | 915 | 931 | 871 | 1004 | Type I |
| CINAU672 | CGTGGTCTTCAAATCTGGCA | GAGGATGGGTGCTAGCTTCT | 144 | 110 | 114 | 165 | Type IV |
| CINAU673 | GCAGAGTGGTGTGATCTTGG | TTTTGTTGAGACTCGCCAGC | 194 | 170 | 205 | 182 | Type IV |
| CINAU674 | GCTGAAACTCCTGATGTGGT | GCCTGTGAGAAAGCGAATGT | 609 | 524 | 487 | 593 | Type II |
| CINAU675 | CTCCTCACCAAGTTCCCCAG | GGTCTTCCATTTTCACACTCAAA | 926 | 936 | 937 | 848 | Type VI |
| CINAU676 | ATCCGCTCCGCCATTCTTAA | GTGGGAGGTAGTAGGCGAAG | 119 | 119 | 116 | 100 | Type II |
| CINAU677 | GCAAAACTTGTACTTCGTGAACT | CCTGTGCCAGCCTTGATTTC | 394 | 406 | 451 | 633 | Type I |
| CINAU678 | ATGCTATTGTTGCTGAGCCG | GTGCACTTCCAAGCTCAAGG | 754 | 723 | 739 | 851 | Type VI |
| CINAU679 | AAGGGCAGGACAAGGTGTAC | CAACTTGGTCTTCTGCGTGG | 224 | 124 | 119 | 104 | Type IV |
| CINAU680 | AAAGGGCTTGGGAATGGGTA | TTCAATGGCAAACAACTTAACCA | 750 | 760 | 781 | 710 | Type II |
| CINAU681 | GTGCTTCGAGACCATGAACC | CGATGTTCCTCTCGCTGTCG | 301 | 298 | 298 | 314 | Type VI |
| CINAU682 | CAGTGAGCTTTTGGGCCATA | TGTTGCCAGATCCTTGCC | 698 | 701 | 691 | 930 | Type VI |
| CINAU683 | CCGATTGTGGAGCCTGAGAT | GTTCATCGCGTTCAGGTTCA | 208 | 177 | 196 | 225 | Type VI |
| CINAU684 | GGCTTCTCTTGGTCTGGGAA | TCCGCATTCTCTTTCCTCGG | 391 | 386 | 386 | 342 | Type VI |
| CINAU685 | TGTTGCAGAGGGAAGGGC | GGCAACCCGTCAGTAACAAG | 276 | 275 | 279 | 440 | Type VI |
| CINAU686 | AGGCTGCAATGAAGAACTGC | TGTTGTTCATCCAAAAGTATGCC | 338 | 350 | 335 | 362 | Type VI |
| CINAU687 | ACAGCTCATCATGCAGGACA | GTCACTGTCTTGAGCAAATGGA | 632 | 545 | 687 | 433 | Type I |
| CINAU688 | GTGGACCTGCTCGTGGAG | AGTCTGATGCCATGATCCTGT | 75 | 75 | 75 | 199 | Type VI |
| CINAU689 | GTGCTCCCAATAACCCATGC | AGGAGGATGACAAAGACTAGCA | 116 | 116 | 117 | 143 | Type VI |
| CINAU690 | TGACTCTTTCAGCATCAGTTCC | GCCATGGGTTGTTGAAGCTA | 210 | 209 | 209 | 291 | Type VI |
| CINAU691 | ACTGACCATCTATGCTCATACGT | GTGCAAGTAATGTCGCAGGA | 397 | 398 | 397 | 367 | Type VI |
| CINAU692 | ACAAGGTCAAGCAGTGCATC | AGTGATCTCCTCCTCCCGG | 233 | 89 | 281 | 105 | Type I |
| CINAU693 | GAGCGCCATGGTGAAATCAT | AGTGAGCAGTCCAGGAGC | 80 | 94 | 80 | 105 | Type VI |
| CINAU694 | CAGTACTTCTCGGCCAAGC | GGACGATGGACATGGACCC | 114 | 225 | 271 | 78 | Type V |
| CINAU695 | CATAGGTTTGTGGCTGCCTG | CTCGATCTTCTCCGGAGCTA | 168 | 173 | 168 | 352 | Type VI |
| CINAU696 | CTGGAAGACGACATTGGCG | CAGCAGGCGGTCGTTGAG | 193 | 427 | 183 | 232 | Type VI |
| CINAU697 | GCTGGGTTGCTGCTTCTTTA | CCGTGATAAACCATTGACTTGC | -357 | 388 | -347 | 243 | Type I |
| CINAU698 | CTATCCCATGCTCCACCACA | GCTAGAACTTTGCGCACCAT | 131 | 111 | 161 | 252 | Type VI |
| CINAU699 | ACCATGCAGATTAGTCAAGGG | GGAGCCCCAAATAAGTCAACC | 225 | 372 | 326 | 200 | Type I |
| CINAU700 | TGTACAGGCTGCAAAACTTCA | CAGTTGTTTGGGTGGCAGAC | 343 | 348 | 350 | 86 | Type VI |
| CINAU701 | CTCTTCACCGCTCCAGACTC | AGAGCCTGTGATGGTCATGA | 550 | 562 | 470 | 441 | Type I |
| CINAU702 | GTGATGCTCCTCCGGCTC | TCGTGTCATTGCCAACGC | 393 | 406 | 642 | 455 | Type V |
| CINAU703 | TCCCCGCCGAACTACAAAAT | TTTACACCCTGAGAGCGTCC | 840 | 832 | 839 | 660 | Type VI |
| CINAU704 | ATCGACCTCCTCATCTGCTG | TCACTCTCATTCCTTACAGCCT | 502 | 647 | 531 | 488 | Type III |
| CINAU705 | CATGGCTTTCGGTACTGGC | CACTTGCTGATGTCGCTGC | 488 | 550 | 525 | 415 | Type VI |
| CINAU706 | AGAGGTGGTGTGTATCTCTCT | GCCAGGCTTTCGATTGCATA | 181 | 181 | 181 | 195 | Type VI |
| CINAU707 | GACAAGTCCTGGCCGAAG | TAGTCCAGTCCACCAACAGC | 313 | 297 | 316 | 273 | Type VI |
| CINAU708 | CTCAACCTCATCCCCATCGT | AGCTTCATCTTTCCATGCCAC | 98 | 110 | 98 | 131 | Type V |
| CINAU709 | CACATCCACTGCATGCAAGT | CTGATGCTGTCGTAGTTGGC | 73 | 69 | 83 | 113 | Type VI |
| CINAU710 | GCATACTCACACTCCAAATTAGC | GGTTCCGATGATTCCAGGGT | 225 | 229 | 230 | 394 | Type VI |
| CINAU711 | TGACGATAAGCATAACTCTGAGG | AAAAGCCTGGTCCAAGGC | 240 | 363 | 232 | 332 | Type IV |
| CINAU712 | AGGTACCTTGCGGAGTTCAA | GCAAGTCCAAGCCTTATCGG | 199 | 200 | 197 | 242 | Type VI |
| CINAU713 | CTGAAGACTGGGAGCTAGCA | TTTGCTGCTCTTCAACCTTTG | 207 | 127 | 117 | 103 | Type II |
| CINAU714 | GGACATCACGCTCCCCTT | TGCTCTCTTAAGTCGGCAGA | 249 | 273 | 143 | 120 | Type II |
| CINAU715 | TGACAGATGGAAAGCATTTGGT | CAGGGACCTCTGGAAGAACT | 135 | 137 | 135 | 91 | Type VI |
| CINAU716 | GGTCGATTTGGAGAATACCCT | GCAGGATCTTGTGGTTCATTGT | 741 | 739 | 740 | 510 | Type IV |
| CINAU717 | CAATCGGAGGGCAAGATGTG | ACCGCTTGTACGTCGGAA | 169 | 167 | 163 | 320 | Type VI |
| CINAU718 | ACCAGGCCCTCATATACAAGT | CCGGGTCAGAATCTGCATTG | 467 | 442 | 353 | 502 | Type VI |
| CINAU719 | GCTCAAGGACATAGATGCTACC | GCTCTTTGGTCATTTTGTTGCA | 136 | 136 | 139 | 125 | Type VI |
| CINAU720 | TTTGAGCCCCTCCATAAGCT | AGCCATCCACATTCCAAATCT | 225 | 235 | 229 | 266 | Type VI |
| CINAU721 | ACACCCGAGCATGATGATACA | CGAAGGTACGTGTCACAGTA | 235 | 208 | 184 | 323 | Type VI |
| CINAU722 | GCTACATTGTTTCCTCCGACA | GCTCACGTCCTCCATCTTGA | 367 | 363 | 356 | 468 | Type VI |
| CINAU723 | GCTCAAATTGAAGACGTACGC | AGACCAGCTCCAGAAAGTGC | 227 | 230 | 195 | 251 | Type II |
| CINAU724 | AGATTGATGCCATGGATGCG | TGAATCCCCTTGCTGGCAA | 654 | 745 | 750 | 627 | Type VI |
| CINAU725 | TGGTGTATGAAGGCCAATCTTG | AGCTTCCTTCAGTCCCCATC | 83 | 83 | 82 | 113 | Type VI |
| CINAU726 | TGTTGTTGAAGCTATGATCGCA | TCTCAAGAAATTCATGCCTGGT | 562 | 383 | 382 | 359 | Type I |
| CINAU727 | TGGAAGTGGCAAAGGTGAAC | CCCTTGTCGCCTCTTGAAAC | 426 | 426 | 414 | 489 | Type VI |
| CINAU728 | GGAGGTGCCTTTGGTAAATGA | CATGCAGTTTACCACCTCCG | 120 | 119 | 118 | 146 | Type VI |
| CINAU729 | AGAACAAAAGCAATGGAGGTCC | CCTCTCCAGTTTTATTGCCTCG | 224 | 102 | 102 | 83 | Type II |
| CINAU730 | GGGTTTGGACTGTCAGCTTG | ACCGGGGTTGATGCTACAAA | 959 | 1074 | 1075 | 691 | Type IV |
| CINAU731 | CGTGACGCTTTGGTATAGAGC | ACCAGGGAAAAGAGCCTGTT | 573 | 573 | 573 | 562 | Type VI |
| CINAU732 | TCAGAGCTGGACGAAACTGA | TATGCTAAGTTCGCCCCTGC | 170 | 178 | 177 | 664 | Type VI |
| CINAU733 | CGCTGTCCCCTTCGTCAC | ATATCAGGAGCAGAACGAGC | 116 | 111 | 115 | 91 | Type VI |
| CINAU734 | CGTCAGGATGATCGAGGAGA | AACATACAGGCCCCTTTCGC | 901 | 970 | 902 | 959 | Type VI |
| CINAU735 | TGAAGATCGTGTTCCTTCCTCT | CATGCTTTCTTCATCCCCTGG | 1583 | 1600 | 939 | 659 | Type V |
| CINAU736 | CCTGGGGCAAGAACAAACAT | TGAACATGGTAAACTCGGTGC | 671 | 6887 | 666 | 395 | Type VI |
| CINAU737 | AAACGAGCTTTGCATGGAGG | CTTTGCATGTTGAGAAGGACAA | 339 | 146 | 340 | 119 | Type III |
| CINAU738 | TGAGGAGTTGCAGCATTTGG | GGTCGTTCTCTTTGCTCAGG | 92 | 92 | 92 | 166 | Type VI |
| CINAU739 | GTTGTAACATTTGGAACTGCTGT | TCATCGAACCACCAAGAAACG | 121 | 138 | 145 | 183 | Type VI |
| CINAU740 | ACGAGGCAAGTTTGATGAAGAG | CCCCTCAGCAGCAACCAATA | 316 | 246 | 268 | 285 | Type I |
| CINAU741 | TGGGAAGAGCGGAAGTTTCT | GGCTTTACACAGTGGACACC | 336 | 339 | 336 | 286 | Type VI |
| CINAU742 | CCTCCTGGGTTTGATTCAAGC | TGACTTGAAGATGGATGTTGCC | 321 | 2140 | 2132 | 259 | Type VI |
| CINAU743 | TGGTTTGCGATACATTGAGGA | TCCCTCTTCTCCATCAACGC | 252 | 253 | 252 | 224 | Type VI |
| CINAU744 | TTCAGAAGACATGCCCCTGT | ACCTTCTCACCCTGCTTCTC | 122 | 113 | 127 | 216 | Type III |
| CINAU745 | GATGATGCCGCTCTTCTTCG | CCGTGCAGAGGATCAGCC | 901 | 917 | 880 | 825 | Type I |
| CINAU746 | CCCATAATGCACGACGGC | TGGTTGATGATCTCCCGCTG | 92 | 82 | 95 | 106 | Type VI |
| CINAU747 | CAGCTCGATGTTTCTTGGCT | GCTGCAATGCTTAGCTCTCC | 526 | 528 | 518 | 543 | Type VI |
| CINAU748 | GCTTTACCGCTTATGTTGCC | TCTGATTCGGTATTTCCTTGCT | 491 | 500 | 496 | 584 | Type VI |
| CINAU749 | CGTGAGTACAAGGCGAAGAAG | GCACTGACATACTCCTCCTCA | 137 | 137 | 135 | 71 | Type VI |
| CINAU750 | GCGATGAAGTTTATGAATGTGGA | AGCGATCTCCTTCTTTCCGT | 238 | 236 | 455 | 210 | Type II |
| CINAU751 | GACAGGAAAAGGAAGCGGTC | CGGATCTTCTTGGCGAACAG | 114 | 103 | 115 | 133 | Type VI |
| CINAU752 | GACAAGGTTGCCAAGGTCAG | CTTCCAGTCCCGCTTCCTAA | 133 | 127 | 127 | 153 | Type VI |
| CINAU753 | GAGGAGAGATGCCGCACC | CTTCACGTTCCCTTTGACGG | 471 | 460 | 415 | 439 | Type VI |
| CINAU754 | GAGCGAATCCTCCATGACGA | CTGTGTGGGATGCAGGAAC | 97 | 94 | 94 | 81 | Type VI |
| CINAU755 | AAGGATTCCGAGGGCAAGG | TTGAGCGGTAGTGATCCTCC | 398 | 404 | 423 | 354 | Type II |
| CINAU756 | TGCTCTTCATCTGCTCCCAC | ACAGCATCTCCGACAGCC | 99 | 105 | 102 | 208 | Type VI |
| CINAU757 | GGCCGAAATCTGATCATGGA | GCAGATCTTCTATGCAAGTAACC | 3457 | 1401 | 1251 | 477 | Type V |
| CINAU758 | AGTTCAGGACAAAGGCTCACT | AAAGCAGGTCTGTGTCGGAT | 789 | 778 | 776 | 677 | Type VI |
| CINAU759 | ACATCAGTGCAGTCAAGAAGTG | GCCTTGATGCATTTGTTGAGC | 953 | 1092 | 949 | 722 | Type IV |
| CINAU760 | GATGGACGTCAGGAGCAAAG | ATGGGTCCTTGAAGATGGCT | 131 | 133 | 143 | 100 | Type VI |
| CINAU761 | GTCAAGATGCAGTCGTCCCT | CGGATACCCTGATGCTTGGA | 205 | 179 | 205 | 237 | Type IV |
| CINAU762 | GTTGTGCAAAGAAGGGCGTA | CCTTATCTGACATGCTCGCG | 310 | 311 | 322 | 256 | Type VI |
| CINAU763 | CGAGGCTGATGGATTTCGTC | TTTAGCCTGCAACTTCTCCG | 99 | 98 | 99 | 89 | Type VI |
| CINAU764 | AGAGGAAAGTAACGGCAAAAGT | GCTTCACCAATGCCCATAACA | 295 | 295 | 293 | 210 | Type IV |
| CINAU765 | GAGCATCAGTCGAGGAAGGA | TGATGACCCTGGCTTTCTCG | 97 | 93 | 94 | 174 | Type VI |
| CINAU766 | GGACAACTTCTTCCACTGCA | TCACCGAGTAGCACGATCC | 107 | 97 | 91 | 75 | Type V |
| CINAU767 | CAACGAGGCGCTCATGTTC | TTGGAGGGGTTGGTGATGAG | 145 | 144 | 139 | 125 | Type VI |
| CINAU768 | CGCTATGTACCGTGAATGGC | CTGGAGAAGTTTAACAGCCAATG | 548 | 546 | 549 | 520 | Type VI |
| CINAU769 | CTCTGCCTACCTTGCTTCCT | AGCAGTTAGAACACAGGTCAA | 142 | 142 | 142 | 125 | Type VI |
| CINAU770 | ACCCACCTTTTCATCCAATGG | TCCTCCGTGTACTCAAGCAA | 511 | 438 | 486 | 405 | Type I |
| CINAU771 | TTCGCCGTCTCCGTCAAC | GGTCTTGATGATCCAGCCCT | 460 | 408 | 475 | 504 | Type IV |
| CINAU772 | AGCTGTCGAAGTATTTGTCATGG | TGAGATGATCCACGGCATGA | 690 | 672 | 663 | 496 | Type VI |
| CINAU773 | AATTGCTTCTCTTGGCGTAAAG | ATGCACATGAACAGCCATCC | 101 | 100 | 100 | 118 | Type VI |
| CINAU774 | GGACTTGATTTGCCCGGC | CCATGACCACTGCACTTGTC | 348 | 342 | 361 | 739 | Type VI |
| CINAU775 | ATGGCCCTGTAGAAGTTGCT | AGTAATCCTCGCCAGCATCA | 295 | 219 | 216 | 255 | Type VI |
| CINAU776 | TGTCGTAGCAGCTTTTGAGG | CCCAGCTGTGTTGTCGATTG | 131 | 134 | 131 | 171 | Type VI |
| CINAU777 | CTGGAGAGGTCATTGGCCT | CGTCACGATGTTTCCGCTTA | 293 | 293 | 292 | 266 | Type VI |
| CINAU778 | GGATACATGCAGCGTTACAAC | GATGGTGCTAGCTGTGAACG | 149 | 104 | 90 | 73 | Type IV |
| CINAU779 | CATGCCCCTTCCTCCTCTAC | TCTGGTGCTCTATGGAAGCG | 670 | 117 | 109 | 117 | Type VI |
| CINAU780 | CTTAGGTGCTGACTGTGTGC | CACCCATCCAACCAGACTGA | 316 | 311 | 324 | 389 | Type VI |
| CINAU781 | CTGGATGCCACATTTTCCGT | TGTCGGTATCAGACTCAAAGGT | 422 | 420 | 422 | 599 | Type II |
| CINAU782 | AGCCCTACTTCCTGGACGT | GAGCTGCACAGGGAACAATC | 326 | 364 | 368 | 397 | Type VI |
| CINAU783 | TTTGTACCAGGGGATCAGGG | GAGAAAGATCCGGCGAGAGA | 159 | 147 | 158 | 186 | Type V |
| CINAU784 | GCAGATCCAAGCAGCACATT | CCTGTCCAACAAGCTCTTCG | 261 | 258 | 265 | 181 | Type VI |
| CINAU785 | GGAGCAGTGGTCTTTATGCA | TGCGAAAGAACTTGAGCTCG | 92 | 92 | 92 | 105 | Type VI |
| CINAU786 | AGGTCATTATACAAGGAATCCGT | GATGGGTGCTTGTCAAATTGA | 647 | 653 | 764 | 382 | Type II |
| CINAU787 | TGTGCACTTGACCAGGTTTG | TCTCTCCACTGCAACCTTCA | 722 | 722 | 723 | 676 | Type VI |
| CINAU788 | GCAACTCAGCAACTCGAACT | CTGTTTATGTGATCTTGTGCCAA | 328 | 255 | 243 | 145 | Type IV |
| CINAU789 | TCCCATACATAGGATTGCTCTTG | AAGAGACCCAAGAGCTCGAG | 126 | 126 | 126 | 87 | Type VI |
| CINAU790 | AGAACCAGAAGCTCGTGGAA | TTGCTTCCTCTTGTCGGAGT | 694 | 689 | 790 | 578 | Type II |
| CINAU791 | TACACGCAAACACAACAGGG | GGTCTCCGGTGAATCTTTCC | 564 | 550 | 556 | 602 | Type VI |
| CINAU792 | TCTTGTGCCCAAACTGCTTT | TCCAGTCCATTCTCTTCCTCTG | 91 | 91 | 89 | 126 | Type VI |
| CINAU793 | GCACCCTCTTAGCACTCCAT | GTGGCCAACTTTTGCTGTAGA | 813 | 829 | 1663 | 846 | Type VI |
| CINAU794 | CGGAAGAAGCTGGAGGAGG | ATTGTTGGCAGAAAGAGCGG | 656 | 720 | 832 | 763 | Type I |
| CINAU795 | CCCTCTGGTTCATAAGGAGGT | GCAGGCAAGAAACTCTGTGT | 324 | 292 | 324 | 344 | Type VI |
| CINAU796 | ATGCCTCGCCGTCTTGAT | GCCGTTAACAGTGAGGATGG | 334 | 378 | 322 | 400 | Type IV |
| CINAU797 | CACCGGGGTTCCACATGAA | CAAAACCAGCTATCACGTCCA | 160 | 160 | 161 | 187 | Type VI |
| CINAU798 | GACGATAATGGAGCACTGCC | CATTGGAGGACGAGGAGGTG | 188 | 187 | 188 | 206 | Type VI |
| CINAU799 | TCTATGTGTAACTGCAGCGC | GGCCTACCTTCTGACTTTGC | 160 | 160 | 160 | 150 | Type VI |
| CINAU800 | CTCGCCTACCTCTACGCC | ATGATCACGTCCGGCTTGTA | 79 | 94 | 140 | 122 | Type VI |
| CINAU801 | GTTCGACGAGTACACCGC | GTGCCCAACCAAAATCACCT | 434 | 502 | 1420 | 301 | Type VI |
| CINAU802 | TGTGAAGGAGAAGCTCTGGA | AGATCCACGCATGTTAAGCA | 631 | 635 | 634 | 589 | Type VI |
| CINAU803 | ATCATACCCTGCAGCGAGAA | AGCTGCTTCACGGTGTACTC | 94 | 94 | 100 | 115 | Type VI |
| CINAU804 | TCCGATCAGAGTTCCCACAC | CATGGAGCCTTTTCAGCCTG | 679 | 913 | 692 | 652 | Type VI |
| CINAU805 | CAAAACCTGCCGGCGTTG | TCTTCAGATTCATCATGGGGTAA | 158 | 166 | 134 | 182 | Type II |
| CINAU806 | ACTCGCGAAACTGACAAACA | AGCGTGTCCTTGGATTCCA | 152 | 157 | 160 | 192 | Type VI |
| CINAU807 | TGAGAAGTACAAAGCCAAGGT | GTCGCATCACCAAGCCTTTG | 559 | 534 | 562 | 520 | Type VI |
| CINAU808 | CGGAAGAAGCTGGAGGAGG | ATTGTTGGCAGAAAGAGCGG | 313 | 418 | 426 | 300 | Type I |
| CINAU809 | CACGATTCTTGAGATTTCCTGGT | CTTAACCATTCGCCTTCAACG | 528 | 528 | 279 | 136 | Type II |
| CINAU810 | CCAACATCAAGAAGGCCACG | CAACAACTGGGCAAACACCT | 221 | 223 | 222 | 235 | Type VI |
| CINAU811 | GGCAACAGACATCGTTATCTCC | TGCAACCGTGAACTCTTTGA | 632 | 572 | 627 | 719 | Type VI |
| CINAU812 | AAGGAGCAGATCGACGAGTT | GATCATCTCCCGCAGCTCC | 115 | 120 | 97 | 83 | Type VI |
| CINAU813 | CCGTGCAGCTACAAAATGGT | CCTGCCGCTCTAACATCTTC | 147 | 146 | 147 | 136 | Type VI |
| CINAU814 | AGACGGCCTACCTGTTCATC | AGAGTCGTTGATGAGGCAGG | 115 | 111 | 106 | 84 | Type VI |
| CINAU815 | CTGTTGGCAAGACAAGGCTG | TTCACACACTTTTCCACGGA | 102 | 101 | 100 | 76 | Type VI |
| CINAU816 | CAGCGACGAGGAAAGTGATG | AGTCTCCCTTTTGCTTCCAGA | 293 | 289 | 150 | 274 | Type VI |
| CINAU817 | AGTGATGACCGCAGACAGAA | GCAACTAAATTTGGCAGGTCC | 118 | 120 | 105 | 132 | Type VI |
| CINAU818 | AGGCAATAGGAGAAGGGCAG | TGCAACCAAAAGAACATACATCA | 138 | 125 | 128 | 193 | Type VI |
| CINAU819 | GGTTCAACAGCCAAGGAGTA | TCATCTCCTCCTCTCCTTCTGA | 878 | 900 | 912 | 752 | Type VI |
| CINAU820 | TTTGCAGCAACATGGAACGT | CCTCGATCACAAGCACGTTC | 567 | 597 | 580 | 612 | Type VI |
| CINAU821 | AGGTCCAAAGAAAGCACATCT | GGATCAACATTTCCCAGAACAAT | 639 | 616 | 558 | 524 | Type VI |
| CINAU822 | CGGTTGCAATCTCGTTTGGA | ACTGTTGCATTTGAAGGGCA | 795 | 809 | 822 | 590 | Type VI |
| CINAU823 | GCAGATCAAGCGGGAAATCG | ATTGTGTCAAAGAGCTCGCC | 242 | 258 | 249 | 290 | Type VI |
| CINAU824 | GCTGTCCTTGCATCACGG | TCCGCAGTTTTAGGATCACTT | 250 | 241 | 251 | 356 | Type VI |
| CINAU825 | GTGTGATGTTCCAGCCGTTC | CGAAGTAGGCGTAGAGGGAG | 201 | 201 | 201 | 227 | Type VI |
| CINAU826 | TGCGGCTGATAGTAGAGAGA | ATTGCCAAGGAACAATCGCA | 1205 | 337 | 342 | 194 | Type II |
| CINAU827 | ACATCATGTGGGATCCTACTGG | TCCATCTCATGAACCGAGGT | 404 | -460 | 338 | -355 | Type VI |
| CINAU828 | GCCACATATTGTCAACTGCG | TCCGGTCCGAGAGAGAATTG | 142 | 142 | 141 | 161 | Type VI |
| CINAU829 | TGCTGCCCCTGAATATGTGA | CAGAAGGACGACGCCAAAG | 192 | 192 | 192 | 205 | Type VI |
| CINAU830 | ACTTTGAAATTAAGAGGTGGGCT | ACGTTGCCTTTCTGTACTGC | 115 | 120 | 115 | 94 | Type VI |
| CINAU831 | TTCCTCCTCCAGACCATCCT | ACAACCACCCTCCATTTGTG | 123 | 122 | 121 | 92 | Type VI |
| CINAU832 | GTGGGAACTGAGTGCAAATTCT | GGGAGATAATCACGTTAAGAGGC | 225 | 320 | 320 | 300 | Type I |
| CINAU833 | GGATGTGCCTGAAACAGTTGA | CCGGCAACAAGAAGTCCCAT | 248 | 775 | 276 | 211 | Type II |
| CINAU834 | GGATGTTCTCTGCCAGTTCC | TGCCATCCATTGTAGACCAGT | 120 | 118 | 118 | 95 | Type VI |
| CINAU835 | TGGGTGACTCTGCCTACAAG | GCAAAGTTTGGAAGGAAGCC | 600 | 630 | 640 | 561 | Type VI |
