# Supplementary material for: Development of intron targeting (IT) markers specific for chromosome arm 4VS of Haynaldia villosa by chromosome sorting and next-generation sequencing
Source: BMC Genomics. 2017 Feb 15;18:167. doi: 10.1186/s12864-017-3567-z (PMC5310052; doi:10.1186/s12864-017-3567-z)
Supplement: Additional file 6: Table S3. — PCR products of 100 IT markers. (DOCX 101 kb) [file 12864_2017_3567_MOESM6_ESM.docx]

| Table S3 PCR products of 100 IT markers | NAU421 | NAU428 | NAU429 | NAU433 | NAU435 | T4VS/4DL |
| --- | --- | --- | --- | --- | --- | --- |
|  | 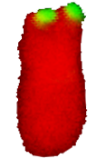 | 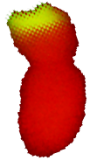 | 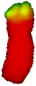 | 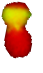 | 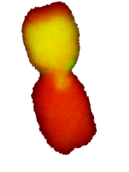 | 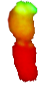 |
| CINAU663 | + | + | + | - | - | + |
| CINAU664 | + | + | + | - | - | + |
| CINAU679 | + | + | + | - | - | + |
| CINAU687 | + | + | + | - | - | + |
| CINAU699 | + | + | + | - | - | + |
| CINAU708 | + | + | + | - | - | + |
| CINAU711 | + | + | + | - | - | + |
| CINAU714 | + | + | + | - | - | + |
| CINAU715 | + | + | + | - | - | + |
| CINAU717 | + | + | + | - | - | + |
| CINAU720 | + | + | + | - | - | + |
| CINAU705 | + | + | + | - | - | + |
| CINAU724 | + | + | + | - | - | + |
| CINAU737 | + | + | + | - | - | + |
| CINAU738 | + | + | + | - | - | + |
| CINAU744 | + | + | + | - | - | + |
| CINAU749 | + | + | + | - | - | + |
| CINAU662 | + | + | + | - | - | + |
| CINAU733 | - | + | + | - | - | + |
| CINAU740 | - | + | + | - | - | + |
| CINAU739 | - | + | + | - | - | + |
| CINAU680 | - | + | + | - | - | + |
| CINAU712 | - | + | + | - | - | + |
| CINAU716 | - | + | + | - | - | + |
| CINAU727 | - | + | + | - | - | + |
| CINAU741 | - | + | + | - | - | + |
| CINAU770 | - | + | + | - | - | + |
| CINAU752 | - | + | + | - | - | + |
| CINAU692 | - | + | + | - | - | + |
| CINAU757 | - | + | + | - | - | + |
| CINAU691 | - | + | + | - | - | + |
| CINAU760 | - | + | + | - | - | + |
| CINAU761 | - | + | + | - | - | + |
| CINAU765 | - | + | + | - | - | + |
| CINAU789 | - | + | + | - | - | + |
| CINAU802 | - | + | + | - | - | + |
| CINAU769 | - | + | + | - | - | + |
| CINAU771 | - | + | + | - | - | + |
| CINAU729 | - | + | + | - | - | + |
| CINAU758 | - | + | + | - | - | + |
| CINAU768 | - | + | + | - | - | + |
| CINAU776 | - | + | + | - | - | + |
| CINAU670 | - | + | + | - | - | + |
| CINAU723 | - | + | + | - | - | + |
| CINAU775 | - | + | + | - | - | + |
| CINAU685 | - | + | + | - | - | + |
| CINAU773 | - | + | + | - | - | + |
| CINAU666 | - | + | + | - | - | + |
| CINAU766 | - | + | + | - | - | + |
| CINAU756 | - | + | + | - | - | + |
| CINAU698 | - | + | + | - | - | + |
| CINAU726 | - | + | + | - | - | + |
| CINAU759 | - | + | + | - | - | + |
| CINAU764 | - | + | + | - | - | + |
| CINAU777 | - | + | + | - | - | + |
| CINAU772 | - | - | + | - | + | + |
| CINAU736 | - | - | + | - | + | + |
| CINAU665 | - | - | + | - | + | + |
| CINAU721 | - | - | + | - | + | + |
| CINAU808 | - | - | + | - | + | + |
| CINAU805 | - | - | + | - | + | + |
| CINAU778 | - | - | + | - | + | + |
| CINAU684 | - | - | + | - | + | + |
| CINAU792 | - | - | + | - | + | + |
| CINAU795 | - | - | + | - | + | + |
| CINAU810 | - | - | + | - | + | + |
| CINAU811 | - | - | + | - | + | + |
| CINAU721 | - | - | + | - | + | + |
| CINAU707 | - | - | + | - | + | + |
| CINAU710 | - | - | - | - | + | + |
| CINAU689 | - | - | - | - | + | + |
| CINAU782 | - | - | - | - | + | + |
| CINAU706 | - | - | - | - | + | + |
| CINAU725 | - | - | - | - | + | + |
| CINAU750 | - | - | - | - | + | + |
| CINAU800 | - | - | - | - | + | + |
| CINAU805 | - | - | - | + | + | + |
| CINAU806 | - | - | - | + | + | + |
| CINAU682 | - | - | - | + | + | + |
| CINAU786 | - | - | - | + | + | + |
| CINAU785 | - | - | - | + | + | + |
| CINAU693 | - | - | - | + | + | + |
| CINAU719 | - | - | - | + | + | + |
| CINAU731 | - | - | - | + | + | + |
| CINAU742 | - | - | - | + | + | + |
| CINAU748 | - | - | - | + | + | + |
| CINAU696 | - | - | - | + | + | + |
| CINAU754 | - | - | - | + | + | + |
| CINAU780 | - | - | - | + | + | + |
| CINAU781 | - | - | - | + | + | + |
| CINAU783 | - | - | - | + | + | + |
| CINAU804 | - | - | - | + | + | + |
| CINAU696 | - | - | - | + | + | + |
| CINAU807 | - | - | - | + | + | + |
| CINAU809 | - | - | - | + | + | + |
| CINAU796 | - | - | - | + | + | + |
| CINAU797 | - | - | - | + | + | + |
| CINAU799 | - | - | - | + | + | + |
| CINAU801 | - | - | - | + | + | + |
| CINAU779 | - | - | - | + | + | + |
